# Sick leave patterns before and after commencement of psychological therapy among individuals with common mental disorders: a register-based, quasi-experimental study

**DOI:** 10.1186/s12888-026-07818-3

**Published:** 2026-01-23

**Authors:** Alexis E. Cullen, Emma Pettersson, Elin Lindsäter, Heidi Taipale, Antti Tanskanen, Ellenor Mittendorfer-Rutz, Magnus Helgesson

**Affiliations:** 1 Division of Insurance Medicine, Department of Clinical Neuroscience, Karolinska Institutet, Stockholm, Sweden; 2 Department of Psychosis Studies, Institute of Psychiatry, Psychology & Neuroscience, King’s College London, London, UK; 3 Academic Primary Care Center, Region Stockholm, Sweden; 4 Center for Psychiatry Research, Department of Clinical Neuroscience, Stockholm Healthcare Services and Karolinska Institutet, Stockholm, Sweden; 5 Division of Psychology, Department of Clinical Neuroscience, Karolinska Institutet, Stockholm, Sweden; 6 Department of Forensic Psychiatry, University of Eastern Finland, Niuvanniemi Hospital, Kuopio, Finland; 7 School of Pharmacy, University of Eastern Finland, Kuopio, Finland; 8 Department of Public Health and Caring Sciences, Uppsala University, Uppsala, Sweden

**Keywords:** Psychotherapy, Work disability, General practice, Depression, Anxiety, Stress-related disorders

## Abstract

**Background:**

Psychological therapies have been shown to reduce sickness absence (SA) among individuals with common mental disorders (CMDs) in clinical trials, but their real-world impact is unclear. To address these knowledge gaps, we compared the likelihood of receiving publicly-financed SA compensation among individuals with CMDs who received at least one session of systematic psychological therapy in primary care and individuals with CMDs who did not receive these treatments.

**Methods:**

Primary healthcare registers were used to identify individuals with CMDs in Region Stockholm who had received psychological therapies (*N* = 12,167) and untreated controls (*N* = 40,517). SA was measured at six-monthly intervals in the two years before and after treatment commencement. Crude and inverse probability weighted (IPW) generalised estimating equation (GEE) models were used to compare the likelihood of having > 14 net SA days (primary outcome) and > 30 and > 90 days (secondary outcomes) at each six-month interval to the period preceding treatment commencement (t0). Crude and IPW models were performed in the treated and control groups separately, with effects at each time-point compared via the ratio of odds ratios (ROR).

**Results:**

In the crude model, the treated group showed significantly greater reductions in the likelihood of having > 14 net SA days at t12, t18, and t24 compared to the control group (ROR: 0.82, 0.80, and 0.79, respectively). However, in the IPW models, the likelihood of receiving > 14 net SA days at t6 relative to t0 was significantly higher in the treated group (ROR: 1.33, 95% CI: 1.25–1.40) with no group differences at t12, t18, or t24. Moreover, the treated group fared significantly worse than the control group in IPW models examining > 30 and > 90 net SA days. In sensitivity analyses, individuals receiving 1–2 sessions showed a greater reduction in the likelihood of having the primary outcome relative to the control group at t12, t18, and t24, whilst those receiving greater doses of psychological therapy (6–12 and > 12 sessions) had a significantly higher likelihood of SA at these timepoints.

**Conclusions:**

Our findings tentatively suggest that psychological therapies delivered in primary care are not associated with a reduction in the likelihood of receiving sick leave in people with CMDs. However, due to the possibility of unmeasured confounders, our findings cannot support causal inferences. Further studies in real-world settings are needed to investigate whether treatment-related factors (e.g., therapeutic focus, quality, and fidelity) modify the effect of primary-care delivered psychological therapies on SA outcomes among people with CMDs.

**Clinical trial number:**

Not applicable.

**Supplementary Information:**

The online version contains supplementary material available at 10.1186/s12888-026-07818-3.

## Background

Around 30% of the world’s population will experience a depressive, anxiety, or stress-related disorder (collectively referred to as common mental disorders: CMDs) at some point during their lives [1], with recent data suggesting that the prevalence of CMDs has increased following the COVID-19 pandemic [2]. By limiting the ability to participate in work, these disorders have an enormous personal, societal, and economic impact. Indeed, CMDs have become leading contributors to long-term sickness absence (SA) and disability pension in Western countries [3–6]. Long-term absence from work can have important consequences for people with CMDs, potentially contributing to risk of unemployment [7], permanent exclusion from the labour market [8, 9], and social exclusion [10]. Reducing SA among individuals with CMDs is therefore an urgent public health priority.

Between 60 and 90% of individuals who present to healthcare services with CMDs are treated exclusively within primary care [11–14]. As such, primary care clinics may offer the best opportunity to deliver psychological interventions to improve work capacity in this population. Studies evaluating the impact of psychological therapies on work outcomes distinguish between interventions with a specific return-to-work (RTW) focus and clinically-focused therapies targeting symptoms. In practice, the distinction may be less clear-cut, with both intervention modalities delivered in parallel. Indeed, in Sweden, RTW coordinators are mandatory within healthcare settings [15]. A meta-analysis of 45 RCTs observed that both forms of therapy were associated with significant reductions in SA days compared to care-as-usual among individuals with CMDs; with effect sizes (Hedge’s g) of 0.18 for RTW-focused interventions and 0.17, 0.12, and 0.17 for cognitive behavioural therapy (CBT), problem-solving therapy, and collaborative care, respectively [16]. In contrast, a Cochrane review [17] found only low-certainty evidence to suggest that work- and clinically-focused interventions alone were more effective in reducing SA days than care-as-usual among individuals with depression (standardised mean differences: 0.39 and 0.15, respectively), with moderate-certainty evidence supporting combined therapies. One consistent finding across these reviews was that neither work- nor clinically-focused interventions improved RTW rates compared to care-as-usual [16, 17], possibly reflecting reduced statistical power to detect differences in this binary outcome.

Current evidence therefore suggests that psychological therapies can reduce SA days, but not RTW rates, among individuals with CMDs; however, important knowledge gaps remain. First, these reviews included interventions delivered across a range of occupational and healthcare settings but did not analyse interventions delivered in primary care settings specifically. Second, RCTs in this field have focused on the duration of SA spells and RTW rates, which are both important outcomes among individuals who are already on sick leave. However, the extent to which psychological therapies can reduce the need for publicly-financed SA compensation (i.e., SA spells that extend beyond durations covered by the employer) is unknown; this is crucial given that public expenditure on SA and disability pension in Europe has risen in recent decades [18]. Finally, whilst RCTs are considered the gold standard for evaluating the treatment effects, a substantial proportion of the RCTs included in previous reviews were rated as having a high risk of bias [16, 17], largely relating to unblinded assessments of self-reported outcomes. Moreover, there is increasing concern regarding the extent to which findings from RCTs can be generalised to real-world settings, known as the “efficacy-effectiveness gap” [19]. Differences in findings between RCTs and real-world studies may in part be attributable to selection bias (patients recruited for RCTs may be less symptomatic, have fewer comorbidities, and be more motivated to engage in therapy), biases associated with subjective outcome measures (i.e., a conscious/unconscious desire by patients and clinicians to show improvement), and deviations from gold-standard treatment protocols when implemented in clinical practice [20]. Using routinely-collected data from national/regional registries may help to overcome some of these challenges and provide a real-world estimation of treatment effects.

The present study aimed to address these knowledge gaps by using the extensive registers available in Sweden. Our aim was to determine whether the likelihood of receiving publicly-financed SA compensation among individuals with CMDs differed in the two years before and after receipt of psychological therapy. Our primary outcome was receiving > 14 net SA days (measured at six-monthly intervals), thresholds of > 30 and > 90 days were examined as secondary outcomes. The Swedish healthcare system distinguishes between ‘systematic’ psychological therapies (SPTs: e.g., CBT and psychodynamic therapy) and ‘non-systematic therapies (e.g., counselling and mindfulness). To align our work with the evidence available from previous RCTs examining clinically-focused interventions (with or without a RTW component), we examined treatments designated as SPTs. To determine whether any changes in SA might be attributable to these treatments specifically, we investigated whether individuals who had not received these therapies showed a similar pattern of SA over the study period and directly compared these groups. We hypothesised that the likelihood of SA would decline in the treated group in the two years after treatment commencement (relative to the six-month period preceding treatment) and that the magnitude of reduction shown in the treated group would be greater than the control group. In sensitivity analyses, we explored the effect of different treatment doses, where individuals in the treated group were further categorised according to the number of SPT sessions attended during the two-year follow-up.

## Methods

### Study design

Data were derived from regional and national registers in Sweden (Table 1), linked by the pseudonymised unique personal identification number. According to current Swedish regulations, the use of national data for research purposes does not require informed consent from individuals held in these registers [21]. We adopted a register-based, quasi-experimental design, which are commonly used in real-world evidence studies utilising routine clinical data to evaluate treatment effects [22]. The study period covered the 1st of January 2014 to the 31st of December 2019, corresponding to the first year when diagnoses were included in the Region Stockholm’s VAL database up to the start of the COVID-19 pandemic. Treatment exposure was defined as any primary care visit where SPT was delivered (see Supplementary Material). We included pre- and post-treatment measures of SA, measured in the two years before and after the first date when SPT was received (designated as t0), and an untreated control group, who were assigned a ‘sham’ t0 date that corresponded to the t0 date of a randomly selected treated case who had their first observed CMD diagnosis in the same year and month as the control (see Supplementary Material for further details). Both groups were free to receive other treatments, including non-systematic psychological therapies and psychotropic medications, during the study period. Inverse-probability weighting (IPW) was used to account for confounding by indication by balancing the distribution of baseline sociodemographic and clinical factors (including exposure to other treatments) across treated and control groups. This method estimates the average treatment effect (ATE) in the entire population [23], defined as the average effect in the population if all individuals received treatment.

**Table 1:** National and regional databases used to define the study population and determine exposure, outcome, and covariate measures

Database	Availability	Variables
Region Stockholm’s VAL database (VAL) [24]	Regional	Primary care diagnoses, psychological therapies administered in primary care
Longitudinal Integrated Database for Health Insurance and Labour Market Studies (LISA) [25]	National	Sociodemographic variables
Micro-Data for Analyses of Social Insurance (MiDAS) [26]	National	Sickness absence and disability pension payments covered by the Swedish Social Insurance Agency
National Patient Register (NPR) [27, 28]	National	Diagnoses received in secondary healthcare (inpatient and outpatient)
Prescribed Drug Register (PDR) [29]	National	Prescribed and dispensed medications (except those administered in hospital)
Cause of Death Register (CoD) [30]	National	Death during the study period

### Study population

**Fig. 1:**
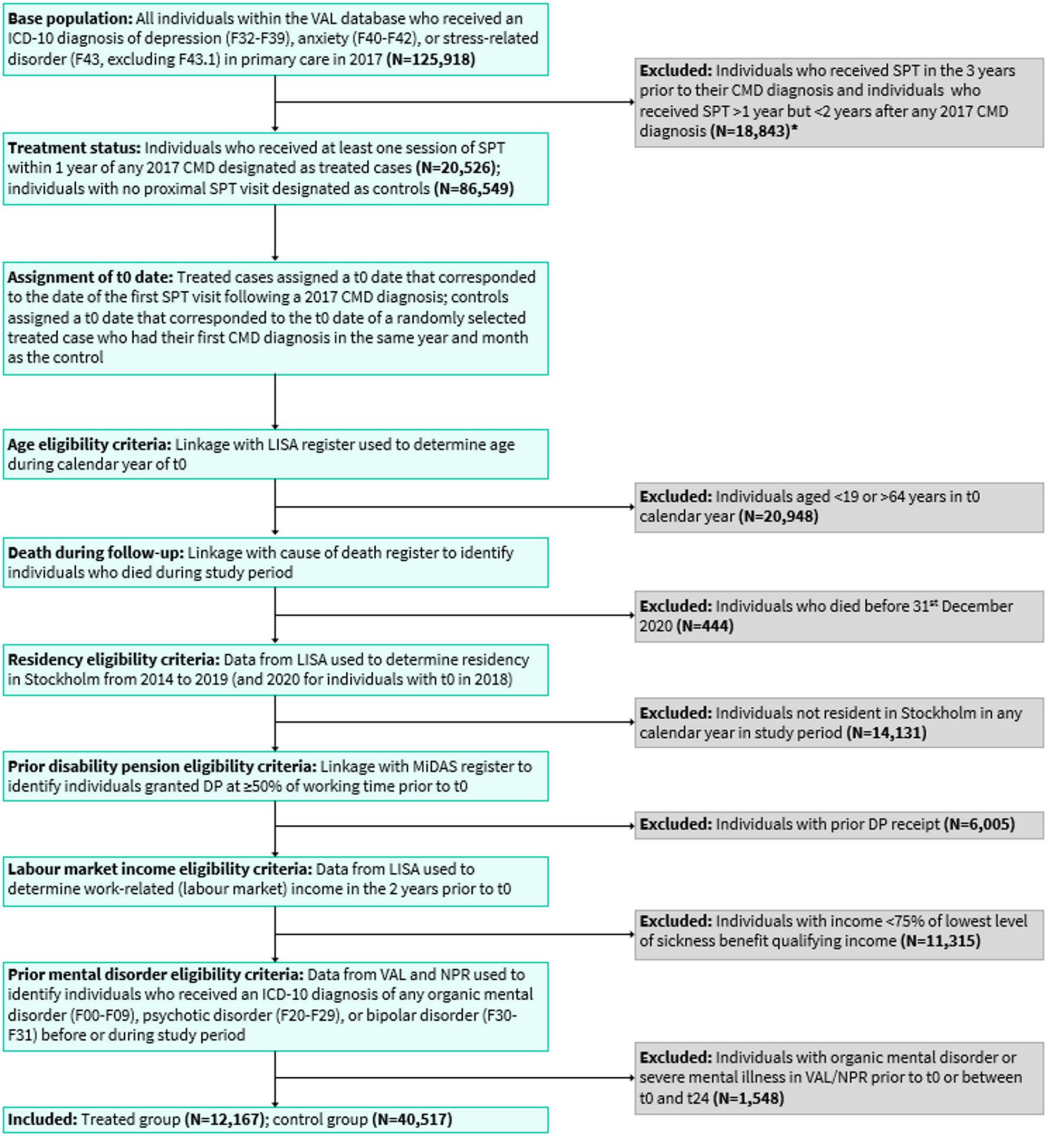
Participant flow through the study. CMD, common mental disorder; DP, disability pension; ICD-10, International Classification of Diseases – version 10; LISA, the Longitudinal Integrated Database for Health Insurance and Labour Market Studies; MiDAS, Micro-Data for Analyses of Social Insurance; NPR, National Patient Register; SPT, systematic psychological therapy; VAL, Region Stockholm’s VAL database

Derivation of the study population is described in detail in Supplementary Material and illustrated in Fig. 1; example cases are provided in Supplementary Fig. 1. The base population included all individuals with at least one diagnosis (according to International Classification of Diseases – version 10: ICD-10 codes [31]) of depression (ICD-10: F32-F39), anxiety (ICD-10: F40-F42), or stress-related disorder (ICD-10: F43, excluding F43.1) in a Region Stockholm primary care clinic during 2017 (*N* = 125,918). Individuals who had received SPT in the three years prior to their 2017 CMD diagnosis were not eligible for inclusion, those who had received at least one session of SPT in the year following any 2017 CMD diagnosis were assigned to the treated group (*N* = 20,526), and those who did not receive SPT in the two relative years after any 2017 CMD diagnosis were designated as controls (*N* = 86,549). Eligible individuals were those aged 19–64 years at t0, who were registered as resident in Region Stockholm during the entire study period, and who were eligible to receive SA benefits. Individuals with prior primary or secondary healthcare contacts for organic mental disorders, psychotic disorders, or bipolar disorder before t0 or in the two relative years thereafter were also excluded as these conditions are likely to be treated in secondary care (and we did not have information on psychological therapy received in these settings) and may be associated with high levels of SA. The final cohort included 12,167 treated individuals and 40,517 untreated controls. The total number of SPT sessions received by the treated group during the two-year follow-up period ranged from 1 to 76; guided by the distribution of the variable (Supplementary Fig. 2) and clinical expertise, we further categorised the treated group according to the number of SPT sessions (1–2 vs. 3–5 vs. 6–12 vs. >12).

### Outcome measure

SA was measured at six-monthly intervals (182 days) in the two years before and after t0. Sweden has a universal social insurance system that provides benefits to workers with reduced work capacity due to disease or injury. As such, all residents aged ≥ 16 years with earnings above a certain level, or who receive unemployment benefits, are eligible for SA benefits. For those in employment, SA payments are covered by the employer for the first 14 days, with the Swedish Social Insurance Agency covering spells > 14 days. For unemployed individuals, the Social Insurance Agency provides payments from day 2 onwards. As we did not exclude individuals who were unemployed at t0, for all individuals, we included only those SA spells > 14 days to reduce potential biases. We calculated net days (length of period * working time extent) such that 20 days of 50% SA payment was converted to 10 net days. For our primary outcome, we derived a binary variable using a threshold of > 14 days (≤ 14 net days vs. >14 net days), thresholds of > 30 net SA days and > 90 net SA days were examined as secondary outcomes. As such, our outcome variables correspond to the six-month period prevalence of having > 14, > 30, and > 90 net days of SA, measured at t-18, t-12, t-6, t0, t6, t12, t18, and t24.

### Covariates

Sociodemographic and clinical variables hypothesised to be associated with both the treatment exposure and the outcome were used to derive IPW model weights in order to account for systematic and chance imbalances in covariates [23]. Full variable definitions and data sources are provided in Supplementary Table 2.

### Statistical analyses

Statistical analyses were performed in R (version 4.3.1) using the ‘WeightIt’, ‘geepack’, ‘cobalt’, and ‘emmeans’ packages. Crude and IPW generalised estimating equation (GEE) models were used to estimate the marginal effect of time on the likelihood of having > 14 net SA days. These models are suitable for longitudinal data that are not normally distributed, and provide more efficient and unbiased estimates than standard regression techniques [32]. IPW model weights were generated using covariate balancing propensity score (CBPS) weighting [33], which simultaneously estimates the propensity scores and ensures covariate balance across groups.

The IPW model incorporated all sociodemographic and clinical covariates using an ATE estimand. To confirm the effectiveness of the weighting strategy, we derived the standardized mean differences and weighted proportion differences for continuous and categorical covariates, respectively, representing the standardised differences between the weighted treated and control groups on these variables. For the weighted samples, we determined the effective sample size (ESS) which represents the size of the unweighted sample that would provide approximately the same level of precision as the weighted sample. GEE models (logit link function) were used to compare the odds of having > 14 net SA days (primary analysis) at each six-month time-point relative to t0, yielding odds ratios (OR) and 95% confidence intervals (CI), in secondary analyses we compared the odds of having > 30 and > 90 net SA days. Models were unbalanced in that individuals were only included at time points where they were able to experience the outcome, that is, those who were granted disability pension or did not quality for SA at a given time point did not contribute to model estimation at that time point. Models were first performed in the treated group and control groups separately (crude and IPW) to estimate the effect of time on SA, estimated effects for each time-point (odds of SA relative to t0) were then compared across groups by deriving the ratio of odds ratios (ROR: OR in treated group/OR in control group) equivalent to a single model with a time*group interaction. Predicted probability plots were generated to visualise the pattern of SA spells in both groups over time. In sensitivity analyses we explored the effect of different treatment doses where individuals in the treated group were categorised according to the number of SPT sessions attended during the two-year follow-up period and compared to the control group.

## Results

### Sample characteristics

Baseline characteristics of the treated (mean age ± SD: 40.5 ± 11.0 years, 72.5% female) and control (mean age ± SD: 42.4 ± 11.3 years, 68.2% female) groups are provided in Table 2. In the crude (unweighted) sample, the treated group had a higher number of primary care visits for CMD than the control group during the two years prior to cohort entry (6.11 ± 7.05 vs. 4.32 ± 5.67) and were far more likely to have received non-systematic psychological therapies in this period (70.4% vs. 36.2). After applying IPW, the standardised differences in the weighted means/proportions between treated and control groups were < 0.001 for all variables (Table 2), indicating that the weighting strategy was highly effective. The treated group was further categorised according to the number of SPT sessions attended during the two-year follow-up: 1–2 (*n* = 6,090, 50.1%), 3–5 (*n* = 2,619, 21.5%), 6–12 (*n* = 2,381, 19.6%), and > 12 (*n* = 1,077, 8.9%). In the crude sample, these subgroups were broadly similar in terms of sociodemographic and clinical characteristics (Supplementary Table 3); however, differences were observed for both the number of primary care visits for CMD (highest in the 6–12 and > 12 SPT session groups) and receipt of non-systematic therapy in primary care (highest in the 1–2 SPT session group) in the two years prior to cohort entry. In the IPW samples (Supplementary Table 4) all standardised differences in weighted means/proportions were reduced to < 0.001.

**Table 2:** Sociodemographic and clinical characteristics of individuals diagnosed with common mental disorders in primary care who received systematic psychological therapies (treated) and those who did not receive these treatments (control) during the study period for the crude and weighted samples

	Crude (unweighted) sample				Inverse probability weighted sample				Standardised difference in weighted means/proportions
	Treated group (*N* = 12,167)		Control group (*N* = 40,517)		Treated group (EES = 36,017 *N* = 53,042)		Control group (EES = 7,235 *N* = 53,042)		
**Age (years)**,** mean (SD)**^**a**^	40.5	(11.0)	42.4	(11.3)	41.83	(11.3)	41.83	(11.3)	< 0.001
**Sex**									
Female	8,827	(72.5)	27,616	(68.2)	36,884	(69.5)	36,884	(69.5)	< 0.001
Male	3,340	(27.5)	12,901	(31.8)	16,158	(30.5)	16,158	(30.5)	< 0.001
**Family situation** ^**b**^									
Married/cohabitant without children	1,315	(10.8)	5,389	(13.3)	6,642	(12.5)	6,642	(12.5)	< 0.001
Married/cohabitant with children	4,252	(34.9)	13,314	(32.9)	17,557	(33.1)	17,557	(33.1)	< 0.001
Single without children	5,451	(44.8)	18,119	(44.7)	23,838	(44.9)	23,838	(44.9)	< 0.001
Single with children	1,149	(9.4)	3,695	(9.1)	5,006	(9.4)	5,006	(9.4)	< 0.001
**Type of residence area** ^**b**^									
Cities	8,706	(71.6)	27,732	(68.4)	36,914	(69.6)	36,914	(69.6)	< 0.001
Towns and suburbs	2,928	(24.1)	10,693	(26.4)	13,633	(25.7)	13,633	(25.7)	< 0.001
Rural areas	533	(4.4)	2,092	(5.2)	2,494	(4.7)	2,494	(4.7)	< 0.001
**Country of birth** ^**b**^									
Sweden	9,895	(81.3)	31,530	(77.8)	41,498	(78.2)	41,498	(78.2)	< 0.001
Rest of the world	2,272	(18.7)	8,987	(22.2)	11,544	(21.8)	11,544	(21.8)	< 0.001
**Level of education** ^**b**^									
≤ 9 years	857	(7.0)	3,668	(9.1)	4,587	(8.7)	4,587	(8.7)	< 0.001
10–12 years	4,596	(37.8)	16,502	(40.7)	21,483	(40.5)	21,483	(40.5)	< 0.001
> 12 years	6,714	(55.2)	20,347	(50.2)	26,972	(50.8)	26,972	(50.8)	< 0.001
**Days of unemployment** ^**b**^									
None	11,415	(93.8)	37,786	(93.3)	49,469	(93.3)	49,469	(93.3)	< 0.001
Any	752	(6.2)	2,731	(6.7)	3,573	(6.7)	3,573	(6.7)	< 0.001
**CMD diagnostic profile** ^**c**^									
Anxiety only	2,889	(23.7)	10,842	(26.8)	13,515	(25.5)	13,515	(25.5)	< 0.001
Depression only	1,520	(12.5)	7,201	(17.8)	8,533	(16.1)	8,533	(16.1)	< 0.001
Stress-related only	2,831	(23.3)	11,059	(27.3)	14,020	(26.4)	14,020	(26.4)	< 0.001
Anxiety and depression	1,164	(9.6)	3,204	(7.9)	4,432	(8.4)	4,432	(8.4)	< 0.001
Anxiety and stress	1,645	(13.5)	3,464	(8.5)	5,363	(10.1)	5,363	(10.1)	< 0.001
Depression and stress-related	1,249	(10.3)	3,013	(7.4)	4,398	(8.3)	4,398	(8.3)	< 0.001
Anxiety, depression, and stress-related	869	(7.1)	1,734	(4.3)	2,781	(5.2)	2,781	(5.2)	< 0.001
**Number of CMD primary care visits**,** mean (SD)**^**c**^	6.11	(7.05)	4.32	(5.67)	5.11	(5.42)	5.11	(8.77)	< 0.001
**Months since first diagnosis**,** mean (SD)**	13.6	(15.0)	16.5	(15.9)	15.97	(16.89)	15.97	(15.49)	< 0.001
**Prior mental disorder diagnoses (non-CMD) - primary care** ^**d**^									
None	1,1619	(95.5)	38,563	(95.2)	50,566	(95.3)	50,566	(95.3)	< 0.001
Any	548	(4.5)	1,954	(4.8)	2,476	(4.7)	2,476	(4.7)	< 0.001
**Prior somatic conditions - primary care** ^**d**^									
None	2,128	(17.5)	7,705	(19.0)	9,855	(18.6)	9,855	(18.6)	< 0.001
Any	10,039	(82.5)	32,812	(81.0)	43,187	(81.4)	43,187	(81.4)	< 0.001
**Prior suicide attempts - primary care** ^**d**^									
None	12,078	(99.3)	40,195	(99.2)	52,621	(99.2)	52,621	(99.2)	< 0.001
Any	89	(0.7)	322	(0.8)	421	(0.8)	421	(0.8)	< 0.001
**Prior receipt of non-systematic therapy – primary care** ^**d**^									
None	3,601	(29.6)	25,854	(63.8)	29,132	(54.9)	29,132	(54.9)	< 0.001
Any	8,566	(70.4)	14,663	(36.2)	23,910	(45.1)	23,910	(45.1)	< 0.001
**Prior mental disorders diagnoses (any) - secondary care** ^**d**^									
None	9,970	(81.9)	31,560	(77.9)	41,672	(78.6)	41,672	(78.6)	< 0.001
Any	2,197	(18.1)	8,957	(22.1)	11,370	(21.4)	11,370	(21.4)	< 0.001
**Prior suicide attempts - secondary care** ^**d**^									
None	12,113	(99.6)	40,285	(99.4)	52,754	(99.5)	52,754	(99.5)	< 0.001
Any	54	(0.4)	232	(0.6)	288	(0.5)	288	(0.5)	< 0.001
**Prior CMD-relevant psychotropic medication** ^**d**^									
None	43,76	(36.0)	11,896	(29.4)	16,707	(31.5)	16,707	(31.5)	< 0.001
Any	7,791	(64.0)	28,621	(70.6)	36,335	(68.5)	36,335	(68.5)	< 0.001
**Prior other psychotropic medication** ^**d**^									
None	11,798	(97.0)	38,300	(94.5)	50,460	(95.1)	50,460	(95.1)	< 0.001
Any	369	(3.0)	2,217	(5.5)	2,582	(4.9)	2,582	(4.9)	< 0.001

CMD: common mental disorder, ESS: effective sample size

^a^ Measured during year of cohort entry

^b^ Measured on 31st December in the calendar year prior to cohort entry

^c^ Measured on cohort entry date and during two relative years (730 days) prior to cohort entry

^d^ Measured in the two relative years (730 days) prior to cohort entry

### Associations of time and treatment status with the primary outcome (> 14 net SA days)

**Fig. 2:**
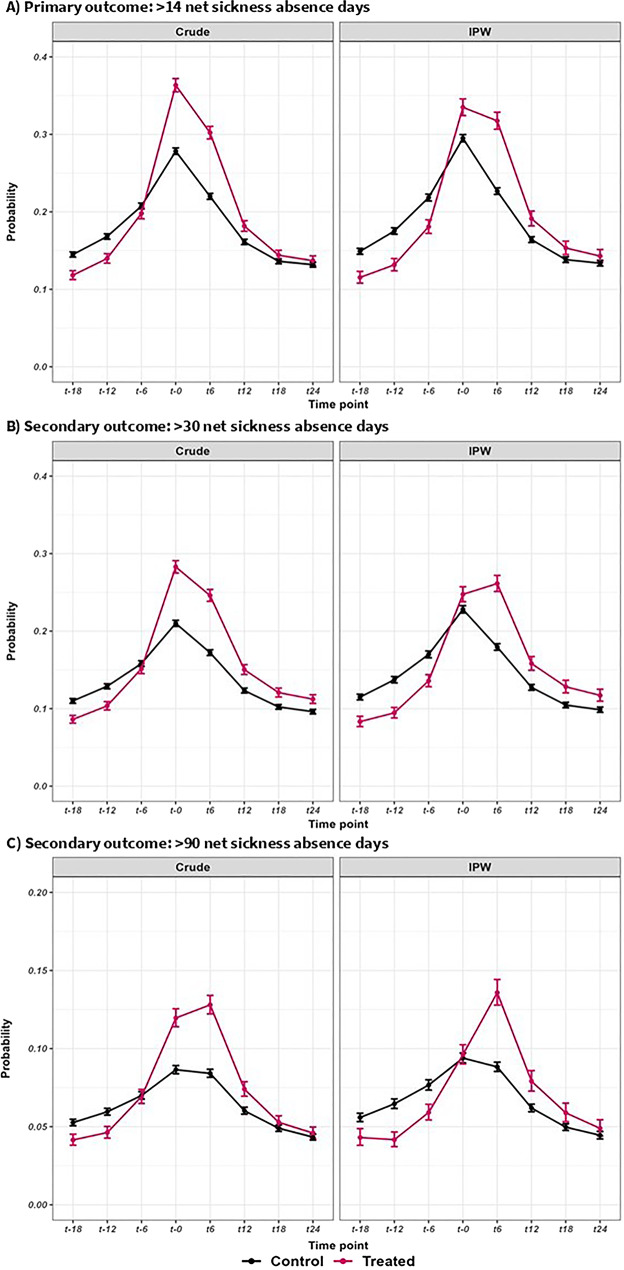
Descriptive statistics for primary and secondary outcomes. Probability (± 95% CI) of having >14 (panel A), >30 (panel B), and >90 (panel C) net sickness absence days at each six-month time interval during the two-years before and after commencement of systematic psychological therapy (t0) among individuals who received at least one therapy session (treated group) and those who did not (control group) in the crude and inverse probability weighted (IPW) samples. Note different y axis scales used across panels

In the crude (unweighted) samples, the probability of having > 14 net SA days during the study period was highest at t0 in both the treated and control groups (Fig. 2, panel A) where 36.4% and 28.0%, respectively experienced the outcome. The crude GEE models (Table 3) indicated that, in both groups, the odds of having > 14 net SA days were significantly lower at all other time periods compared to t0. In the comparison analysis, the likelihood of having SA at t6 compared to t0 was higher (but not statistically significant) in the treated group compared to the control group whereas the odds were significantly lower in the treated group at t12, t18, and t24 (ROR: 0.82, 0.80, and 0.79, respectively). The overall pattern of SA in the IPW sample (Fig. 2, panel A) was broadly similar to the crude sample, although the differences between treated and control groups at t0 were less marked (33.6% and 29.8%, respectively had > 14 net SA days). The GEE models performed in the IPW treated and control groups separately showed similar patterns to the crude models (Table 3). However, in the comparison analysis, the likelihood of having SA at t6 relative to t0 was significantly higher in the treated group than in the control group (ROR: 1.33, 95% CI: 1.25–1.40) and there were no group differences at t12, t18, or t24 (ROR: 1.04, 1.02, and 1.00, respectively).

**Table 3:** General estimating equation (GEE) models examining the odds of having > 14 net sickness absence days (primary outcome) and having > 30 and > 90 net sickness absence days (secondary outcomes) at each six-month time interval relative to the six months prior to t0 among individuals with common mental disorders who received at least one session of systematic psychological therapy (treated group) and those who did not receive any sessions (control group)

Time interval ^a^	Crude model						Inverse probability weighted model ^b^					
	Treated (Tx)		Control (Ct)		Comparison (Tx/Ct)		Treated (Tx)		Control (Ct)		Comparison (Tx/Ct)	
	OR	95% CI	OR	95% CI	ROR	95% CI	OR	95% CI	OR	95% CI	ROR	95% CI
**Outcome (primary outcome): >14 net SA days**												
t-18	**0.24**	**(0.22–0.25)**	**0.43**	**(0.42–0.44)**	**0.55**	**(0.51–0.59)**	**0.26**	**(0.24–0.28)**	**0.41**	**(0.39–0.42)**	**0.63**	**(0.58–0.69)**
t-12	**0.28**	**(0.27–0.30)**	**0.51**	**(0.50–0.53)**	**0.55**	**(0.52–0.59)**	**0.30**	**(0.28–0.32)**	**0.50**	**(0.48–0.51)**	**0.60**	**(0.55–0.65)**
t-6	**0.43**	**(0.41–0.45)**	**0.67**	**(0.65–0.69)**	**0.65**	**(0.61–0.68)**	**0.44**	**(0.41–0.46)**	**0.66**	**(0.64–0.68)**	**0.66**	**(0.62–0.71)**
t0	Ref	---	Ref	---	Ref	---	Ref	---	Ref	---	Ref	---
t6	**0.76**	**(0.73–0.79)**	**0.72**	**(0.71–0.74)**	1.05	(1.00–1.09)	**0.92**	**(0.88–0.97)**	**0.70**	**(0.68–0.71)**	**1.33**	**(1.25–1.40)**
t12	**0.40**	**(0.38–0.42)**	**0.49**	**(0.47–0.50)**	**0.82**	**(0.77–0.87)**	**0.48**	**(0.45–0.51)**	**0.46**	**(0.44–0.47)**	1.04	(0.97–1.12)
t18	**0.32**	**(0.30–0.33)**	**0.40**	**(0.39–0.41)**	**0.80**	**(0.75–0.85)**	**0.38**	**(0.35–0.41)**	**0.37**	**(0.36–0.39)**	1.02	(0.94–1.10)
t24	**0.30**	**(0.29–0.32)**	**0.38**	**(0.37–0.39)**	**0.79**	**(0.74–0.85)**	**0.35**	**(0.33–0.38)**	**0.35**	**(0.34–0.37)**	1.00	(0.92–1.08)
**Outcome (secondary outcome): >30 net SA days**												
t-18	**0.24**	**(0.22–0.26)**	**0.46**	**(0.45–0.48)**	**0.51**	**(0.48–0.56)**	**0.28**	**(0.25–0.30)**	**0.44**	**(0.42–0.46)**	**0.63**	**(0.57–0.69)**
t-12	**0.29**	**(0.27–0.31)**	**0.56**	**(0.54–0.58)**	**0.53**	**(0.49–0.57)**	**0.32**	**(0.29–0.34)**	**0.54**	**(0.52–0.56)**	**0.59**	**(0.54–0.65)**
t-6	**0.45**	**(0.43–0.48)**	**0.71**	**(0.69–0.73)**	**0.64**	**(0.60–0.68)**	**0.48**	**(0.45–0.51)**	**0.69**	**(0.67–0.71)**	**0.69**	**(0.64–0.74)**
t0	Ref	---	Ref	---	Ref	---	Ref	---	Ref	---	Ref	---
t6	**0.83**	**(0.79–0.86)**	**0.78**	**(0.76–0.80)**	**1.06**	**(1.00–1.11)**	**1.08**	**(1.01–1.14)**	**0.74**	**(0.72–0.76)**	**1.45**	**(1.36–1.55)**
t12	**0.45**	**(0.42–0.47)**	**0.53**	**(0.51–0.55)**	**0.85**	**(0.80–0.90)**	**0.57**	**(0.53–0.62)**	**0.49**	**(0.48–0.51)**	**1.16**	**(1.06–1.26)**
t18	**0.35**	**(0.33–0.37)**	**0.43**	**(0.41–0.44)**	**0.81**	**(0.76–0.87)**	**0.45**	**(0.41–0.48)**	**0.40**	**(0.38–0.41)**	**1.13**	**(1.04–1.23)**
t24	**0.32**	**(0.30–0.34)**	**0.40**	**(0.39–0.42)**	**0.80**	**(0.74–0.86)**	**0.40**	**(0.37–0.44)**	**0.37**	**(0.36–0.39)**	1.09	(1.00–1.19)
**Outcome (secondary outcome): >90 net SA days**												
t-18	**0.32**	**(0.29–0.35)**	**0.58**	**(0.55–0.61)**	**0.55**	**(0.50–0.62)**	**0.42**	**(0.36–0.48)**	**0.56**	**(0.53–0.60)**	**0.74**	**(0.64–0.86)**
t-12	**0.36**	**(0.33–0.39)**	**0.66**	**(0.63–0.69)**	**0.54**	**(0.49–0.60)**	**0.40**	**(0.36–0.46)**	**0.65**	**(0.62–0.69)**	**0.62**	**(0.55–0.71)**
t-6	**0.55**	**(0.51–0.59)**	**0.79**	**(0.76–0.82)**	**0.70**	**(0.64–0.75)**	**0.59**	**(0.54–0.64)**	**0.79**	**(0.75–0.83)**	**0.74**	**(0.67–0.82)**
t0	Ref	---	Ref	---	Ref	---	Ref	---	Ref	---	Ref	---
t6	**1.08**	**(1.01–1.15)**	**0.96**	**(0.93–1.00)**	**1.12**	**(1.04–1.21)**	**1.47**	**(1.36–1.60)**	**0.92**	**(0.89–0.96)**	**1.60**	**(1.46–1.75)**
t12	**0.61**	**(0.57–0.66)**	**0.68**	**(0.65–0.71)**	**0.90**	**(0.83–0.99)**	**0.83**	**(0.75–0.92)**	**0.64**	**(0.61–0.67)**	**1.30**	**(1.16–1.46)**
t18	**0.48**	**(0.44–0.52)**	**0.57**	**(0.54–0.60)**	**0.84**	**(0.76–0.92)**	**0.66**	**(0.59–0.74)**	**0.53**	**(0.50–0.56)**	**1.26**	**(1.11–1.42)**
t24	**0.43**	**(0.39–0.46)**	**0.50**	**(0.48–0.53)**	**0.85**	**(0.77–0.94)**	**0.57**	**(0.51–0.64)**	**0.47**	**(0.44–0.49)**	**1.23**	**(1.08–1.39)**

SA, sickness absence; OR, odds ratio; ROR, ratio of odds ratio (OR in treated group / OR in control group); CI, confidence interval

^a^ Time interval corresponds to 6-month intervals (182 days) measured relative to the date of cohort entry (treated group: cohort entry date corresponds to date of first observed systematic psychological therapy; control group: cohort entry date corresponds to randomly selected date from grid of possible start dates based on year and month of first observed CMD visit)

^b^ Inverse probability weights derived using covariate balancing propensity score weighting methods, incorporating all measured sociodemographic and clinical covariates

### Associations of time and treatment status with secondary outcomes (> 30 and > 90 net SA days)

Figure 2 (panel B and C) shows the proportion of individuals having > 30 and > 90 net SA days at each time-point; in the crude samples, 28.3% of the treated group and 21.0% of the control group experienced > 30 SA days at t0 with 12.0% and 8.7%, respectively having > 90 SA days at t0. Results from the GEE models performed in the crude samples were broadly similar to those observed for > 14 days (Table 3) except that for both the > 30 and > 90 SA outcomes, the treated group were significantly more likely to receive SA at t6 relative to t0 compared to controls but significantly less likely to receive SA at t12, t18, and t24 compared to t0. However, in the IPW models, the treated group had significantly higher odds than the control group of having > 30 SA days at t6, t12, and t18 (ROR: 1.45, 1.16. and 1.13) relative to t0 but not at t24, and for > 90 SA days, the treated group had significantly higher odds of having the outcome at t6, t12, t18, and t24 (ROR: 1.60, 1.30, 1.26, and 1.23).

### Sensitivity analyses exploring the effect of treatment dose

**Fig. 3:**
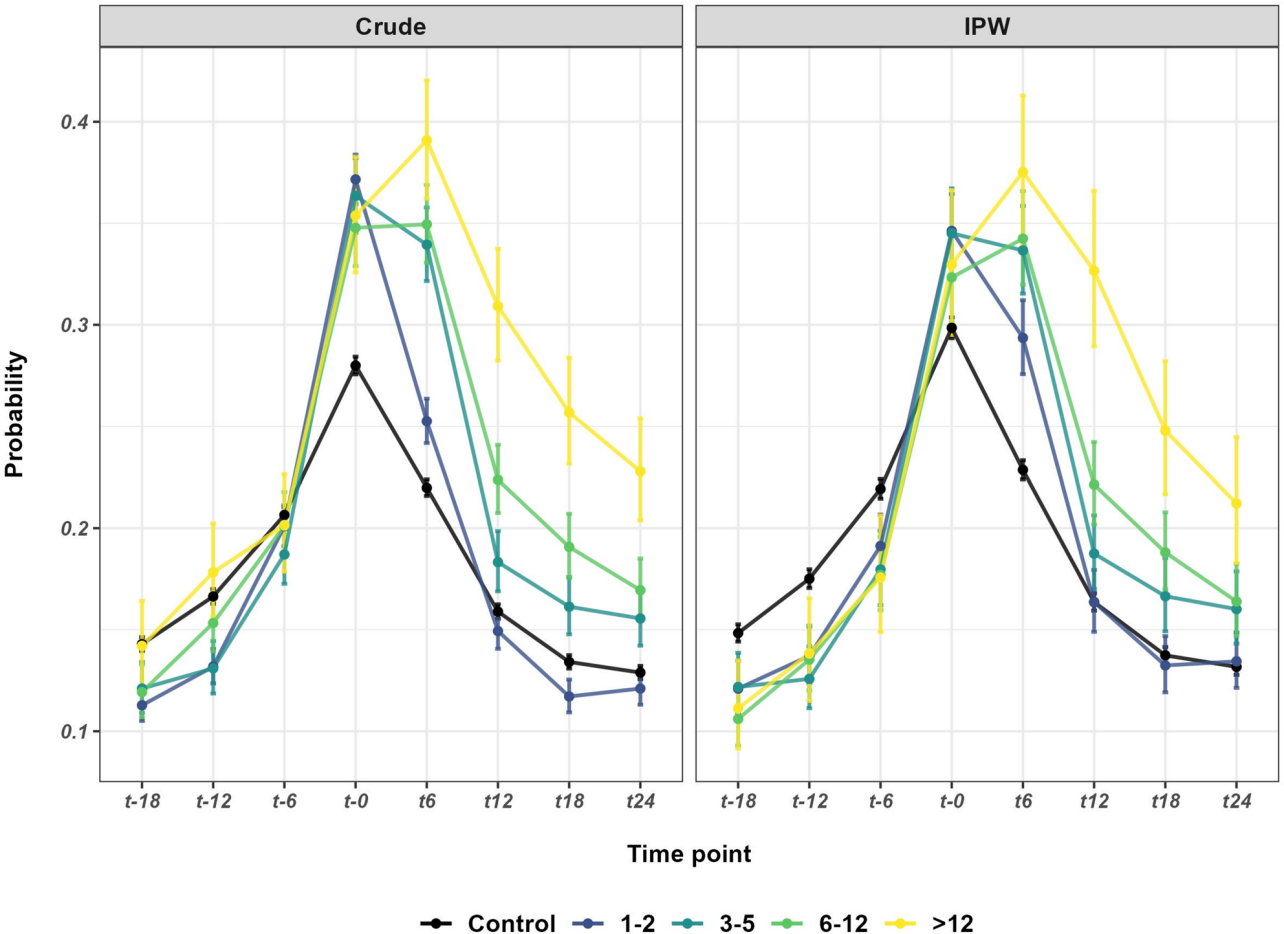
Descriptive statistics for primary outcome by treatment dose. Probability (± 95% CI) of having > 14 net sickness absence days at each six-month time interval during the two-years before and after commencement of systematic psychological therapy (t0) among individuals who received 1–2, 3–5, 6–12, and > 12 therapy sessions (treated group) and those who received no sessions (controls) in the crude and inverse probability weighted (IPW) samples

The probabilities of having > 14 net SA days are shown for all treatment dose groups and the control group in Fig. 3; descriptive statistics are provided for secondary outcomes (> 30 and > 90 SA day thresholds) in Supplementary Table 5. In GEE models (performed for the primary outcome only), the pattern of results varied by SPT dose in both the crude and IPW models (Table 4). Individuals who received 1–2 SPT sessions had significantly higher odds of SA than the control group at t6 in the IPW model (ROR: 1.13) but significantly lower odds at t12, t18, and t24 (ROR: 0.80, 0.77, and 0.82, respectively) whilst those who received 3–5 sessions had significantly higher odds of SA than the control group at t6 relative to t0 (ROR: 1.38) but did not differ at t12, t18, or t24. In contrast, individuals who received 6–12 sessions had significantly higher odds than the control group at t6, t12, and t18 relative to t0 (ROR: 1.57, 1.29, and 1.30), but no significant differences at t24, whilst those who received the highest dose of treatment (> 12 sessions) had significantly higher odds at t6, t12, t18, and t24 (ROR: 1.75, 2.14, 1.79 and 1.54, respectively).

**Table 4:** General estimating equation (GEE) models examining the odds of having > 14 net sickness absence days at each six-month time interval relative to the six months prior to t0 among individuals with common mental disorders who received 1–2, 3–5, 6–12, and > 12 sessions of systematic psychological therapy (treated group) and comparisons with individuals who did not receive any sessions (controls)

Time interval ^a^	Crude model				Inverse probability weighted model ^b^			
	Treated (Tx)		Comparison (Tx/Ct)		Treated (Tx)		Comparison (Tx/Ct)	
	OR	95% CI	ROR	95% CI	OR	95% CI	ROR	95% CI
**Exposure (sensitivity analysis): 1–2 SPT sessions**								
t-18	**0.22**	**(0.20–0.24)**	**0.50**	**(0.46–0.55)**	**0.26**	**(0.23–0.30)**	**0.63**	**(0.56–0.73)**
t-12	**0.26**	**(0.24–0.28)**	**0.50**	**(0.46–0.55)**	**0.30**	**(0.26–0.34)**	**0.60**	**(0.53–0.68)**
t-6	**0.43**	**(0.40–0.45)**	**0.64**	**(0.59–0.68)**	**0.45**	**(0.40–0.50)**	**0.68**	**(0.61–0.75)**
t0	Ref	---	Ref	---	Ref	---	Ref	---
t6	**0.57**	**(0.54–0.60)**	**0.79**	**(0.74–0.84)**	**0.78**	**(0.72–0.86)**	**1.13**	**(1.03–1.24)**
t12	**0.30**	**(0.28–0.32)**	**0.61**	**(0.56–0.66)**	**0.37**	**(0.33–0.42)**	**0.80**	**(0.71–0.91)**
t18	**0.22**	**(0.21–0.24)**	**0.56**	**(0.51–0.62)**	**0.29**	**(0.25–0.33)**	**0.77**	**(0.67–0.88)**
t24	**0.23**	**(0.21–0.25)**	**0.61**	**(0.56–0.67)**	**0.29**	**(0.26–0.33)**	**0.82**	**(0.72–0.94)**
**Exposure (sensitivity analysis): 3–5 SPT sessions**								
t-18	**0.24**	**(0.21–0.28)**	**0.56**	**(0.49–0.64)**	**0.26**	**(0.23–0.31)**	**0.64**	**(0.55–0.75)**
t-12	**0.26**	**(0.23–0.30)**	**0.51**	**(0.45–0.59)**	**0.27**	**(0.24–0.32)**	**0.55**	**(0.47–0.64)**
t-6	**0.40**	**(0.36–0.45)**	**0.60**	**(0.54–0.67)**	**0.42**	**(0.36–0.47)**	**0.63**	**(0.55–0.72)**
t0	Ref	---	Ref	---	Ref	---	Ref	---
t6	**0.90**	**(0.83–0.97)**	**1.24**	**(1.15–1.34)**	0.96	(0.88–1.06)	**1.38**	**(1.26–1.52)**
t12	**0.39**	**(0.35–0.44)**	**0.81**	**(0.72–0.91)**	**0.44**	**(0.38–0.50)**	0.95	(0.83–1.09)
t18	**0.34**	**(0.30–0.38)**	**0.85**	**(0.75–0.95)**	**0.38**	**(0.33–0.44)**	1.01	(0.88–1.17)
t24	**0.32**	**(0.29–0.36)**	**0.85**	**(0.75–0.96)**	**0.36**	**(0.31–0.42)**	1.01	(0.87–1.18)
**Exposure (sensitivity analysis): 6–12 SPT sessions**								
t-18	**0.25**	**(0.22–0.29)**	**0.59**	**(0.51–0.68)**	**0.25**	**(0.21–0.29)**	**0.61**	**(0.51–0.72)**
t-12	**0.34**	**(0.30–0.38)**	**0.66**	**(0.58–0.75)**	**0.33**	**(0.28–0.38)**	**0.66**	**(0.56–0.77)**
t-6	**0.47**	**(0.42–0.52)**	**0.71**	**(0.63–0.79)**	**0.45**	**(0.39–0.52)**	**0.68**	**(0.59–0.78)**
t0	Ref	---	Ref	---	Ref	---	Ref	---
t6	1.01	(0.93–1.09)	**1.39**	**(1.28–1.51)**	1.09	(0.99–1.20)	**1.57**	**(1.41–1.73)**
t12	**0.54**	**(0.49–0.60)**	1.11	(0.99–1.24)	**0.60**	**(0.52–0.68)**	**1.29**	**(1.13–1.48)**
t18	**0.44**	**(0.39–0.50)**	1.11	(0.98–1.25)	**0.48**	**(0.42–0.56)**	**1.30**	**(1.12–1.50)**
t24	**0.38**	**(0.34–0.43)**	1.01	(0.89–1.14)	**0.41**	**(0.35–0.48)**	1.15	(0.99–1.34)
**Exposure (sensitivity analysis): >12 SPT sessions**								
t-18	**0.30**	**(0.25–0.37)**	**0.71**	**(0.58–0.86)**	**0.25**	**(0.20–0.33)**	**0.62**	**(0.48–0.80)**
t-12	**0.40**	**(0.33–0.48)**	**0.77**	**(0.64–0.93)**	**0.33**	**(0.26–0.41)**	**0.65**	**(0.52–0.82)**
t-6	**0.46**	**(0.39–0.54)**	**0.69**	**(0.59–0.81)**	**0.43**	**(0.35–0.53)**	**0.66**	**(0.54–0.81)**
t0	Ref	---	Ref	---	Ref	---	Ref	---
t6	**1.17**	**(1.04–1.32)**	**1.62**	**(1.43–1.83)**	**1.22**	**(1.06–1.41)**	**1.75**	**(1.52–2.02)**
t12	**0.82**	**(0.71–0.94)**	**1.68**	**(1.46–1.94)**	0.99	(0.82–1.18)	**2.14**	**(1.78–2.58)**
t18	**0.63**	**(0.54–0.74)**	**1.58**	**(1.35–1.86)**	**0.67**	**(0.57–0.79)**	**1.79**	**(1.50–2.13)**
t24	**0.54**	**(0.46–0.64)**	**1.42**	**(1.20–1.68)**	**0.55**	**(0.46–0.65)**	**1.54**	**(1.28–1.84)**

SA, sickness absence; SPT, systematic psychological therapy; OR, odds ratio; ROR, ratio of odds ratio (OR in treated group / OR in control group); CI, confidence interval

a Time interval corresponds to 6-month intervals (182 days) measured relative to the date of cohort entry (treated group: cohort entry date corresponds to date of first observed systematic psychological therapy; control group: cohort entry date corresponds to randomly selected date from grid of possible start dates based on year and month of first observed CMD visit)

b Inverse probability weights derived using covariate balancing propensity score weighting methods, incorporating all measured sociodemographic and clinical covariates

## Discussion

This quasi-experimental study examined the association between receipt of systematic psychological therapy and sickness absence in a sample of 52,684 individuals diagnosed with CMDs in primary care and explored the effect of treatment dose. Whilst the crude (unweighted) analyses suggested a positive effect of receiving at least one SPT session on the likelihood of having > 14 net SA days (primary outcome) at 12-, 18- and 24-months after treatment commencement, these effects were not maintained in the IPW model. Moreover, the treated group showed significantly smaller reductions in the likelihood of receiving SA at 6-, 12-, and 18- months after treatment commencement relative to t0 in IPW models when we examined longer periods of SA (> 30 and > 90 net SA days). In IPW sensitivity analyses, we additionally observed that higher treatment doses (6–12 and > 12 sessions) were associated with a greater likelihood of having SA in the two years after treatment commencement relative to the control group. The weighted analyses, which can be used to approximate the causal effect of treatment in observational studies [34], tentatively suggest that individuals with CMDs who receive SPTs in primary care do not show a greater reduction in the likelihood of receiving publicly-financed sickness absence compensation compared to individuals who do not receive SPTs.

Our primary aim was to determine whether individuals with CMD who received any SPT, regardless of the number of sessions, showed a greater reduction in the likelihood of receiving publicly-financed SA when compared to individuals with CMD who did not receive any SPT sessions. Whilst the treated group did show a reduction in the probability of having > 14 net SA days at each 6-month timepoint after treatment commencement, the magnitude of reduction was not significantly different to that observed in the control group at 12-, 18-, and 24-months after treatment commencement in the IPW model, and at 6-months post commencement, the control group showed a greater reduction relative to baseline. Moreover, when we examined longer SA periods (> 30 and > 90 days), the likelihood of having these outcomes at 6-, 12-, and 18-months compared to t0 was significantly higher in the treated group in the IPW models. One possible explanation for these findings is that physicians may be hesitant to terminate sick-leave spells when individuals are actively receiving SPTs and/or may place individuals on sick leave to enable them to focus on treatment. Thus, even though sickness absence compensation should strictly be based on an individual’s capacity to work in relation to disease or injury, in clinical practice, it is possible that sick leave may actually be maintained/initiated to improve therapeutic alliance and support people with CMDs to engage with SPTs. In support of this hypothesis, we observed that those who received higher treatment doses had poorer outcomes than those who received fewer SPT sessions. In fact, individuals who received minimal therapy (1–2) sessions showed greater reductions in the likelihood of having > 14 SA days relative to the control group at 12-, 18-, and 24-months treatment in the IPW model (thus appearing to benefit from treatment), whereas those who received > 12 sessions showed significantly smaller reductions (i.e., higher ROR) at all timepoints after treatment commencement. Although these findings were unexpected, they are consistent with those observed in a recent register-based observational study from Finland [35] which found that individuals with depression and anxiety who received shorter durations of psychotherapy showed greater reductions in the likelihood of receiving SA than those with longer treatment durations. This pattern of findings may be confounded by illness severity/complexity (which were unable to adequately capture in the present study), which is likely associated with both dose of treatment and the probability of receiving SA.

Weighting was used to balance the treated and control groups on important sociodemographic and clinical variables, some of which may influence the likelihood of receiving treatment (i.e., confounding by indication). As such, the IPW models are intended to provide a closer approximation of the causal effect of SPT on SA than standard regression models which adjust for potential confounders [23, 34]. However, these methods require several assumptions to be met, including (i) that the model is correctly specified, (ii) that all covariates related to treatment exposure and the outcome are used to derive the weights, with no unmeasured confounders, and (iii) that all individuals have the possibility to receive/not receive treatment. Concerning the first assumption, the CBPS method used to derive the IPWs is relatively robust to model misspecification [33] and was highly effective in this sample. Moreover, the treated and control groups were more closely matched on the risk on the primary outcome (> 14 net SA days) at t0 in the weighted sample and we achieved even closer matching when we examined longer durations of SA (to the extent that there were no differences in the proportion of individuals having > 90 net SA days at t0 after weighting). With regards to the second point, although the weights were generated from a wide range of sociodemographic and clinical variables that we hypothesised to be related to both exposure status and the outcome based on our previous research [36–38], there may be unmeasured confounders. Importantly, Region Stockholm’s VAL database does not include information on illness severity (e.g., symptoms and functioning), and so we were unable to control for the possibility that the treated group were more unwell/impaired, which may have contributed to the increased likelihood of SA in this group at t6 relative to the control group. Indeed, in the crude samples, the treated group was more likely to receive all three CMD diagnoses (depression, anxiety, and stress-related) and had a higher number of previous CMD primary care visits than the control group, both of which suggest that the treated group had more severe/complex presentations than the control group. As noted above, differences in illness severity may also explain the pattern of findings by treatment dose, as individuals with more severe symptoms may require a greater dose of treatment, which is supported by the fact that these same CMD variables were associated with treatment dose. Third, Stockholm County is a large region which includes the capital city of Stockholm, several other cities and towns, and large rural areas [39]; hence, the availability of SPT may vary across individual clinics. As such, it is unknown whether all individuals had the possibility to receive treatment. For these reasons, whilst we attempted to estimate the causal effect of SPT on SA using robust methodological approaches, the findings from this real-world study may not approximate those derived from RCTs.

Several additional limitations should be noted. First, our study population was heterogeneous with respect to diagnosis in that we included individuals with depression, anxiety, and stress-related disorders. Whilst it is not uncommon for RCTs in this field to include mixed samples (reflecting the high levels of comorbidity across these CMDs), some have observed differential effects of treatment when results are stratified by diagnosis [40, 41]. Second, as this was a real-world study, the treated and control groups were free to receive other treatments throughout the study period, and it is possible that these treatments influenced SA. However, previous studies investigating the effect of antidepressants [42, 43] and non-systematic psychological therapies [44, 45] on SA among people with CMDs have yielded null or mixed findings, suggesting that these treatments are unlikely to have had a major impact on SA patterns. Third, whilst we were able to identify types of psychological therapies administered in primary care by their administrative code, and only included those designated as systematic (e.g., CBT), we have no information on the specific content/focus of treatment. As such, we were unable to determine whether individual sessions focused on symptoms and/or promoting return to work or assess the quality and fidelity of SPTs delivered in primary care irrespective of the focus of treatment. Fourth, it is important to acknowledge that our outcome variable — publicly-financed SA — is to a large extent dictated by social insurance policies [46]. As such, the patterns that we observed may be due to structural factors and financial incentives rather than an effect of any treatment. Finally, the findings from this study may have limited generalisability to other countries, particularly those without publicly financed healthcare systems. Due to these limitations, our results must be interpreted with caution.

Our findings contrast with those observed in RCTs where psychological therapies have been found to reduce SA days among individuals with CMDs [16, 17, 47]. This difference in findings is somewhat expected and likely reflects the complexities of evaluating interventions in real-world settings where multiple factors, including illness severity, patient preference, and resources, are likely to influence which individuals receive treatment, which types of treatment they receive, and the duration of treatment. Determining the real-world effect of psychological treatment on SA is further complicated by the fact that the physicians who are responsible for in managing SA and RTW decisions also make recommendations regarding treatments; for example, a physician may prescribe SA and recommend that the patient also receives psychological therapy, and may to some extent adjust SA duration in line with therapeutic progress/scope. As noted above, this may increase the likelihood that SA is initiated/maintained to facilitate engagement in therapy. Taken together, our findings suggest that SPT does not reduce the likelihood of having SA, and those who receive higher doses (> 6 sessions) may even have a higher likelihood of SA than individuals who receive no treatment. However, it is important to note that psychological therapies may have led to improvements in clinical outcomes (e.g., symptom reduction, quality of life, relapse prevention, or reduced need for secondary care CMDs [48–50]), as such, provision of these treatments in primary care may have broader benefits for individuals with CMDs that were not captured in this study. Moreover, it is possible that it may take longer to observe an effect of psychological treatment on SA; indeed, a recent RCT evaluating psychological therapies in people with depression and/or anxiety found that improvements in work-related outcomes were only observed in longer follow-ups (> 2 years after treatment [51]) whereas significant improvements in depression and anxiety symptoms were observed at the 6-month follow-up [52]. Follow-up beyond two years may be necessary to observe a positive effect of SPT on SA.

## Conclusions

In this large naturalistic, quasi-experimental study, we observed that individuals with CMDs who received SPT in primary care showed a significant reduction in their likelihood of having > 14 net SA days at 12-, 18-, and 24-months after treatment commencement when compared to the six months prior to treatment. However, the same pattern was observed in a control group who did not receive SPT, and after accounting for differences in sociodemographic and clinical characteristics at baseline, the magnitude of reduction in the likelihood of SA at these time-points did not differ in the treated and control groups. Furthermore, individuals who received higher doses of SPT (> 6 sessions) had poorer outcomes than those who received 1–2 SPT sessions, the latter in fact showed significant reductions in the likelihood of receiving SA compared to the control group in the IPW analyses. Our findings therefore tentatively suggest that SPTs delivered in primary care may have limited impact on public expenditure on sick leave. However, it is important to note that whilst IPW models were used to estimate causal effects, we cannot rule out the possibility that the groups differ on important unmeasured confounders that may have influenced treatment receipt/dose and our outcome. Given the potential risk of confounding by indication, we are unable to determine whether the treated group would have experienced poorer SA outcomes than those observed had they not received psychological treatment.

## Supplementary Information

Below is the link to the electronic supplementary material.

Supplementary Material 1

## Data Availability

The data used in this study cannot be made publicly available due to privacy regulations. According to the General Data Protection Regulation, the Swedish law SFS 2018:218, the Swedish Data Protection Act, the Swedish Ethical Review Act, and the Public Access to Information and Secrecy Act, these types of sensitive data can only be made available for specific purposes, including research, that meets the criteria for access to this sort of sensitive and confidential data as determined by a legal review. Readers may contact Professor Ellenor Mittendorfer-Rutz (Ellenor.mittendorfer-rutz@ki.se) regarding the data.
