# Supplementary material for: Sick leave patterns before and after commencement of psychological therapy among individuals with common mental disorders: a register-based, quasi-experimental study
Source: BMC Psychiatry. 2026 Jan 23;26:87. doi: 10.1186/s12888-026-07818-3 (PMC12849155; doi:10.1186/s12888-026-07818-3)
Supplement: Supplementary file 1 — Supplementary Material 1 [file 12888_2026_7818_MOESM1_ESM.docx]

**Supplementary Material**

Cullen AE et al. Sick leave patterns before and after commencement of psychological therapy among individuals with common mental disorders: A register-based, quasi-experimental study.

## Systematic psychological therapy

Receipt of any systematic psychological therapy in primary care was determined using Region Stockholm’s VAL database (VAL). All visits to primary care recorded within this database are coded according to:

- Date of visit
- Diagnosis (1-8 per visit) coded according to the International Classification of Diseases – version 10 (ICD-10)
- Classification of Health Interventions (Klassifikation av vårdåtgärder: KVÅ) codes (1-10 per visit), defined according to the Swedish classification of care measures (KVÅ: <https://www.socialstyrelsen.se/globalassets/sharepoint-dokument/dokument-webb/klassifikationer-och-koder/kva-inledning-och-beskrivning-2015.pdf>)
- Healthcare provider (1 to 5 per visit)
- Type of contact (1 per visit), including information on whether the visit occurred

Systematic psychological therapies and their respective action codes are provided in Table S1. In addition, receipt of other (non-systematic) psychological therapies in the two years prior to cohort entry was included as a covariate (used to derive inverse probability weights), defined as any other visit (irrespective of action code) where the healthcare provider was a psychologist, psychotherapist, or ‘curator or any visit with a ‘curator’ action code (XS913).

**Table S1.** Systematic psychological therapy KVÅ codes

| **Action code** | **Treatment type** |
| --- | --- |
| DU008 | Systematic psychological treatment, psychodynamic (PDT) |
| DU009 | Systematic psychological treatment, other |
| DU010 | Systematic psychological treatment, cognitive |
| DU011 | Systematic psychological treatment, cognitive behavioural therapy (CBT) |
| DU013 | Systematic psychological treatment, mentalization based (MBT) |
| DU014 | Eye movement desensitisation reprogramming (EMDR) |
| DU020 | Systematic psychological treatment, systemic |
| DU021 | Systematic psychological treatment, dialectical behaviour therapy (DBT) |
| DU022 | Systematic psychological treatment, interpersonal therapy (IPT) |

For both systematic and non-systematic psychological therapies, we excluded visits where no patient contact occurred (e.g., a visit was scheduled but the patient did not attend) as determined using the ‘type of contact’ variable. If a visit included more than one relevant action code/caregiver type, each was coded separately.


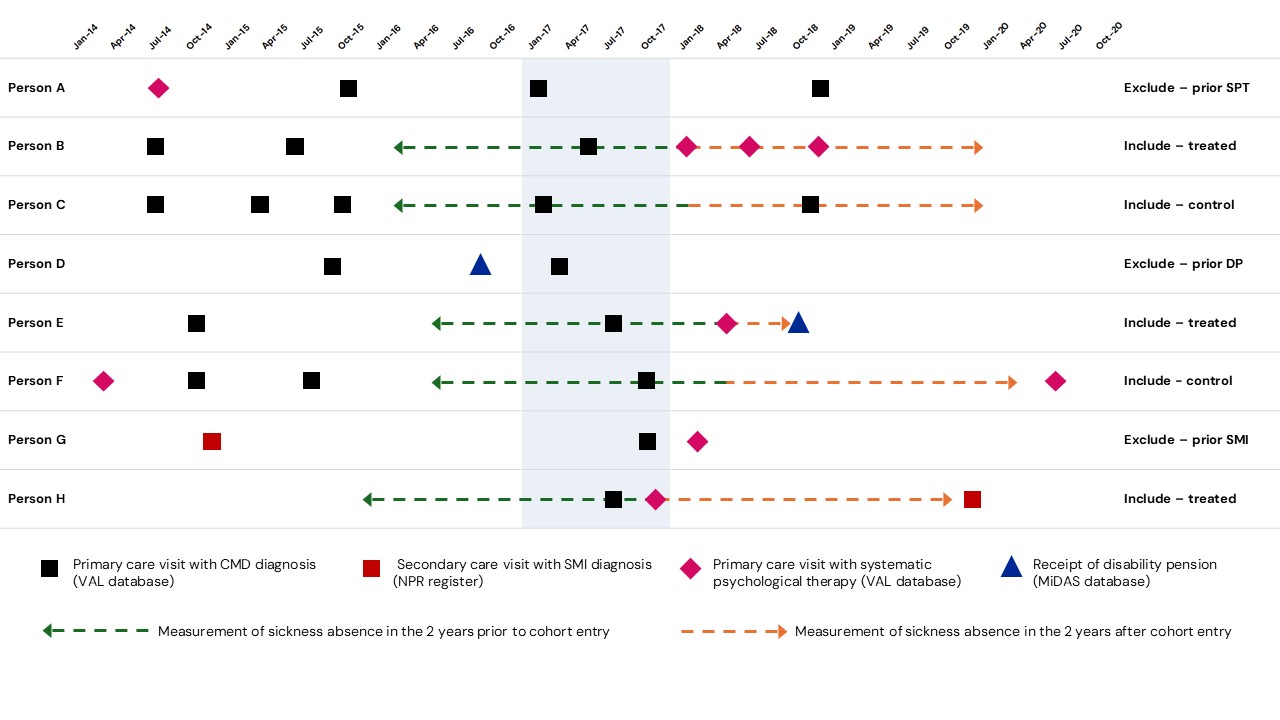


**Figure S1.** Determination of inclusion and treatment status. CMD, common mental disorder; t0, start date; SPT, systematic psychological therapy; DP, disability pension; SMI, severe mental illness. Individuals with a primary care visit with a CMD diagnosis in 2017 are assessed for eligibility. Person A is excluded as they received SPT in the 3 years prior to their 2017 CMD visit. Person B received SPT proximal to their 2017 CMD visit and is included as a treated case, their t0 date corresponds to the date of their first proximal SPT visit. Person C is included as a control, they are assigned a t0 date that matches a randomly selected treated case (in this example, person B) who had their first observed CMD diagnosis in the same month and year as person C’s first observed CMD diagnosis (July 2014). Person D received DP at 50% or more in the year prior to their 2017 CMD diagnosis and is therefore excluded. Person E is included as a treated case as they received SPT within 1 year of their 2017 CMD visit but is censored at the point when they receive DP as they are no longer able to experience sickness absence. Person F is included as a control as they did not receive SPT until 3 years after their 2017 CMD visit, they are assigned a t0 date (based on month and year of their first observed CMD diagnosis) that matches person E. Person G is excluded as they received a prior diagnosis of SMI in secondary care. Person H is included as treated case as they did not receive an SMI diagnosis until 2 years after t0.


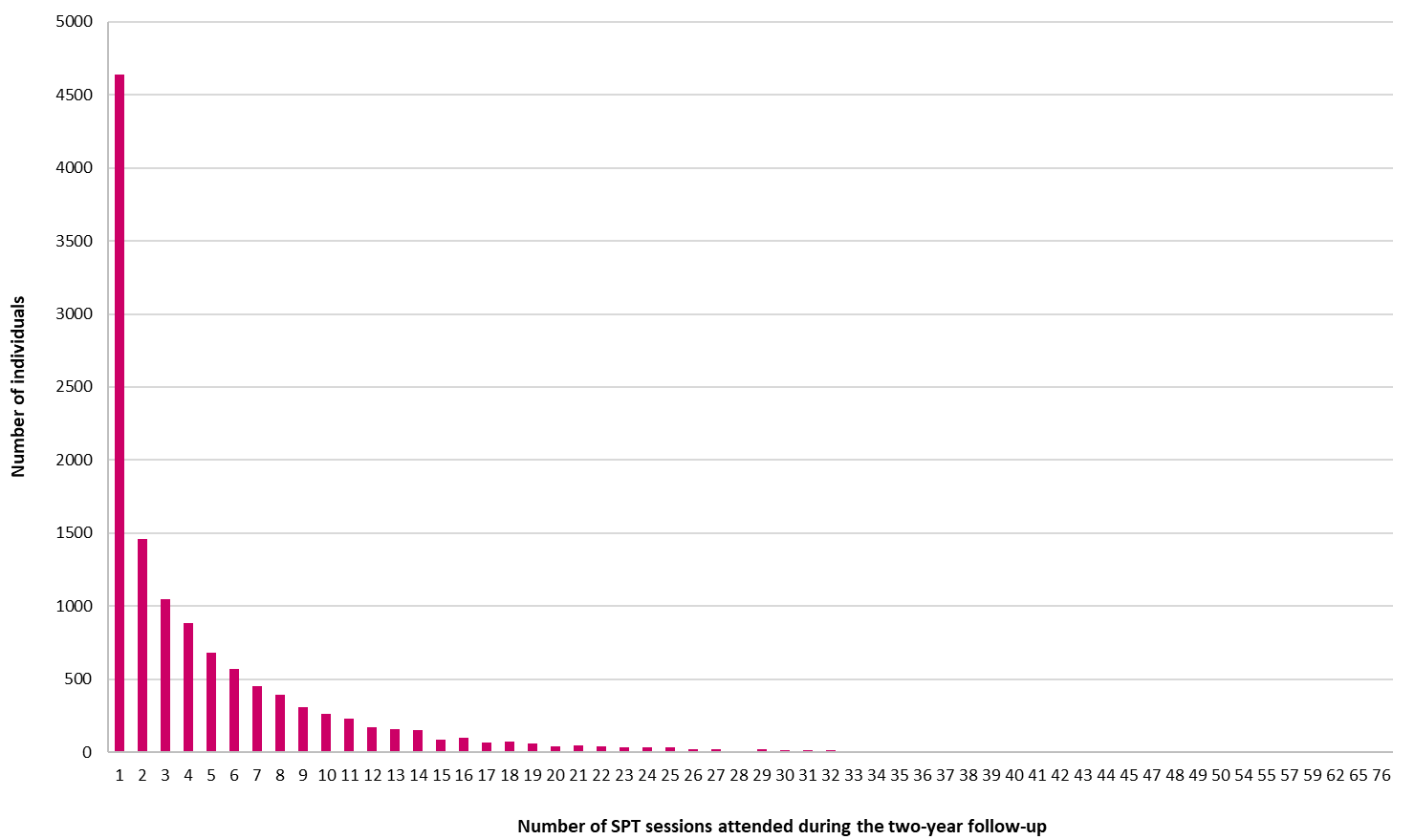


**Figure S2.** Number of systematic psychological therapy (SPT) sessions attended by individuals in the treated group during the two years after treatment commencement. Treatment dose subsequently categorised as 1-2 vs. 3-5 vs. 6-12 vs. >12 sessions.

**Table S2.** Variable definitions and data sources

| **Variable** | **Type** | **Definition** | **Register** |
| --- | --- | --- | --- |
| Common mental disorder | Inclusion variable | Diagnosis of any depressive (ICD-10 codes F32-F39), anxiety (ICD-10 codes F40-F42), or stress-related (ICD-10 code F43, excluding F43.1) disorder in primary care during 2017, categorised as no vs. yes | VAL |
| Prior receipt of systematic psychological therapy | Exclusion variable | Any visit to primary care in the 3 relative years (1095 days) prior to a CMD diagnosis in 2017, where a systematic psychological therapy action code was recorded, categorised as no vs. yes | VAL |
| Treatment status | Exposure variable | Receipt of at least one systematic psychological therapy session in primary care that occurs within 1 relative year (365 days) after any 2017CMD diagnosis, categorised as control vs. treated | VAL |
| Start date (t0) | Other variable | Treated group: date of first systematic psychological therapy that occurs proximal to a 2017 CMD diagnosis; control group: date that corresponds to the t0 date of a randomly selected treated case who had their first observed CMD diagnosis in the same year and month as the control | VAL |
| Age | Exclusion variable | Age (years) during the calendar year of cohort entry, categorised as 19-64 vs. <19 or >64 | LISA |
| Death during follow-up | Exclusion variable | Death after t0, categorised as no vs. yes | CDR |
| Living in Stockholm County | Inclusion variable | Registered as a resident in Stockholm County on the 31^st^ of December each calendar year from 2013 to 2019 inclusive, ^a^ categorised as no vs. yes | LISA |
| Prior receipt of disability pension | Exclusion variable | Disability pension granted at ≥50% of working time in any year prior to cohort entry, categorised as no vs. yes | MiDAS |
| Labour market income | Inclusion variable | Work-related (labour market) income in the calendar year prior to cohort entry <75% of the lowest level of sickness benefit qualifying income (24% of price base amount ^b^), categorised as no vs. yes | LISA |
| Organic disorders | Exclusion variable | Diagnosis of any organic mental disorder (ICD-10 codes F00-F09) in primary or secondary care prior to cohort entry or during 2-year follow-up period, categorised as no vs. yes | VAL, NPR |
| Severe mental disorders | Exclusion variable | Diagnosis of any psychotic disorder (ICD-10 codes F20-F29) or bipolar disorder (ICD-10 codes F30-F31) in primary or secondary care prior to cohort entry or during 2-year follow-up period, categorised as no vs. yes | VAL, NPR |
| Receipt of disability pension during follow-up | Censoring variable | Receipt of DP during any 6-month time interval such that the individual is unable to experience >14 net days of SA or would not qualify for SA (i.e., >168 net days of DP), categorised as no vs. yes | MiDAS |
| Sickness absence | Outcome variable | Registered as having >14 net SA days, measured during each 6-month (182 day) time-interval relative to t0, categorised as no vs. yes | MiDAS |
| Time interval | Other variable | Time interval (t) relative to cohort entry date, corresponding to 6-month (182 day) periods during which outcome measured, categorised as t-4 vs. t-3 vs. t-2 vs. t-1 vs. t0 vs. t+1 vs. t+2 vs. t+3 | NA |
| Age | Covariate | Age (years) during calendar year of cohort entry | LISA |
| Sex | Covariate | Measured at birth, categorised as female vs. male | LISA |
| Family situation | Covariate | Measured on the 31st of December in the calendar year prior to cohort entry, categorised as married/cohabiting without children vs. married/cohabiting with children vs. single without children vs. single with children | LISA |
| Type of residence area | Covariate | Measured on the 31st of December in the year prior to cohort entry, categorised as cities vs. towns/suburbs vs. rural, according to EUROSTAT’s degree of urbanization (DEGURBA) classification of local administrative units | LISA |
| Country of birth | Covariate | Categorised as Sweden vs. other | LISA |
| Level of education | Covariate | Measured on the 31st of December in the calendar year prior to cohort entry, categorised as ≤9 years vs. 10-12 years vs. >12 years | LISA |
| Unemployment | Covariate | Number of unemployment days during the calendar year prior to cohort entry, categorised as none vs. any | LISA |
| CMD diagnostic profile | Covariate | Includes all CMD diagnoses received in primary care during the 2 relative years (730 days) prior to cohort entry (inclusive of cohort entry date), categorised as anxiety only vs. depression only vs. stress-related only vs. anxiety and depression vs. anxiety and stress-related vs. depression and stress-related vs. anxiety, depression, and stress-related | VAL |
| Number of CMD primary care visits | Covariate | Count of all visits to primary care during the 2 relative years (730 days) prior to cohort entry (inclusive of cohort entry date), where at least one CMD diagnosis was received | VAL |
| Time since first CMD diagnosis | Covariate | Number of months between first recorded CMD visit in primary care and cohort entry date | VAL |
| Prior primary care contact for other (non-CMD) mental disorder | Covariate | Any other (non-CMD) mental disorder diagnosis (all ICD-10 F codes excluding F32-F39, F40-F42, and F43) received in primary care in the 2 relative years (720 days) prior to cohort entry, categorised as no vs. yes | VAL |
| Prior primary care contact for any somatic condition | Covariate | All somatic disorder/condition diagnoses (ICD-10 codes A-Z, excluding F00-F99) received in primary care in the 2 relative years (730 days) prior to cohort entry, categorised as no vs. yes | VAL |
| Prior primary care contact for suicide attempt | Covariate | Any primary care visit for suicide attempt or event of undetermined intent (ICD-10 codes X60-X84, Y10-Y34) during the 2 relative years (730 days) prior to cohort entry, categorised as no vs. yes | VAL |
| Prior receipt of non-systematic psychological therapy in primary care | Covariate | Any primary care visit during the 2 relative years (730 days) prior to cohort entry where a non-systematic psychological therapy was received, categorised as no vs. yes | VAL |
| Prior secondary care contact for any mental disorder | Covariate | Any inpatient admission or specialist outpatient visit with a main or side diagnosis of any mental disorder (ICD-10 codes F00-F99) during the 2 relative years (720 days) prior to cohort entry, categorised as no vs. yes | NPR |
| Prior secondary care contact for suicide attempt | Covariate | Any inpatient admission or specialist outpatient visit for suicide attempt or event of undetermined intent (ICD-10 codes X60-X84, Y10-Y34) during the 2 relative years (730 days) prior to cohort entry, categorised as no vs. yes | NPR |
| Prior dispensation of any CMD-relevant psychotropic medication | Covariate | Dispensations of any psychotropic medication typically used to treat CMDs, including antidepressants, anxiolytics, hypnotics and sedatives, and antihistamines (ATC codes N06A, N05B, N05C, and R06AD01) measured during the 2 relative years (730 days) prior to cohort entry, categorised as no vs. yes | PDR |
| Prior dispensation of any other psychotropic medication | Covariate | Dispensations of any other psychotropic medications, including antipsychotics, antiepileptics, lithium, psychostimulants, drugs used for alcohol dependence, drugs used for opioid dependence, and antidepressants in combination with psycholeptics (ATC codes N05A, N03AF01, N03AG01, N03AX09, N05AN01, N06B, N07BB, N07BC, and N06CA) measured during the 2 relative years (730 days) prior to cohort entry, categorised as no vs. yes | PDR |

ATC, Anatomic Therapeutic Chemical Classification System; CDR, Cause of Death Register; CMD, common mental disorder; DP, disability pension; ICD-10, International Classification of Diseases – version 10; LISA, Longitudinal Integration Database for Health Insurance and Labor Market Studies; MiDAS, Micro-Data for Analyses of Social Insurance; NA, not applicable; NPR, National Patient Register; PDR, Prescribed Drug Register; SA, sickness absence; VAL, Region Stockholm’s VAL database.

^a^ Treated individuals whose cohort entry date was in 2018 were also required to be living in Stockholm County on the 31^st^ of December 2020.

**Table S3. Sociodemographic and clinical characteristics of individuals diagnosed with common mental disorders in primary care who received 1-2, 3-5, 6-12, and >12 sessions of systematic psychological therapies during the two-year follow-up period (treated) and any sessions (control) during the study period for the crude (unweighted) samples**

|  | **Crude (unweighted) sample** | | | | | | | | | |
| --- | --- | --- | --- | --- | --- | --- | --- | --- | --- | --- |
|  | **Control group**  **(N=****40,517)** | | **1-2 SPT sessions**  **(N=6090)** | | **3-5 SPT sessions (N=2619)** | | **6-12 SPT sessions (N=2381)** | | **>12 SPT sessions (N=1077)** | |
|  | **N** | **(%)** | **N** | **(%)** | **N** | **(%)** | **N** | **(%)** | **N** | **(%)** |
| **Age (years), mean (SD) ^a^** | 42.4 | (11.3) | 40.8 | (11.2) | 40.1 | (10.9) | 40.4 | (10.9) | 40.7 | (10.6) |
| **Sex** |  |  |  |  |  |  |  |  |  |  |
| Female | 27616 | (68.2) | 4291 | (70.5) | 1902 | (72.6) | 1764 | (74.1) | 870 | (80.8) |
| Male | 12901 | (31.8) | 1799 | (29.5) | 717 | (27.4) | 617 | (25.9) | 207 | (19.2) |
| **Family situation ^b^** |  |  |  |  |  |  |  |  |  |  |
| Married/cohabitant without children | 5389 | (13.3) | 662 | (10.9) | 279 | (10.7) | 278 | (11.7) | 96 | (8.9) |
| Married/cohabitant with children | 13314 | (32.9) | 2087 | (34.3) | 955 | (36.5) | 837 | (35.2) | 373 | (34.6) |
| Single without children | 18119 | (44.7) | 2805 | (46.1) | 1117 | (42.6) | 1042 | (43.8) | 487 | (45.2) |
| Single with children | 3695 | (9.1) | 536 | (8.8) | 268 | (10.2) | 224 | (9.4) | 121 | (11.2) |
| **Type of residence area ^b^** |  |  |  |  |  |  |  |  |  |  |
| Cities | 27732 | (68.4) | 4296 | (70.5) | 1923 | (73.4) | 1718 | (72.2) | 769 | (71.4) |
| Towns and suburbs | 10693 | (26.4) | 1455 | (23.9) | 608 | (23.2) | 592 | (24.9) | 273 | (25.3) |
| Rural areas | 2092 | (5.2) | 339 | (5.6) | 88 | (3.4) | 71 | (3.0) | 35 | (3.2) |
| **Country of birth ^b^** |  |  |  |  |  |  |  |  |  |  |
| Sweden | 31530 | (77.8) | 4932 | (81.0) | 2110 | (80.6) | 1971 | (82.8) | 882 | (81.9) |
| Rest of the world | 8987 | (22.2) | 1158 | (19.0) | 509 | (19.4) | 410 | (17.2) | 195 | (18.1) |
| **Level of education ^b^** |  |  |  |  |  |  |  |  |  |  |
| ≤9 years | 3668 | (9.1) | 457 | (7.5) | 209 | (8.0) | 139 | (5.8) | 52 | (4.8) |
| 10-12 years | 16502 | (40.7) | 2319 | (38.1) | 1015 | (38.8) | 890 | (37.4) | 372 | (34.5) |
| >12 years | 20347 | (50.2) | 3314 | (54.4) | 1395 | (53.3) | 1352 | (56.8) | 653 | (60.6) |
| **Days of unemployment ^b^** |  |  |  |  |  |  |  |  |  |  |
| None | 37786 | (93.3) | 5703 | (93.6) | 2457 | (93.8) | 2246 | (94.3) | 1009 | (93.7) |
| Any | 2731 | (6.7) | 387 | (6.4) | 162 | (6.2) | 135 | (5.7) | 68 | (6.3) |
| **CMD diagnostic profile ^c^** |  |  |  |  |  |  |  |  |  |  |
| Anxiety only | 10842 | (26.8) | 1520 | (25.0) | 603 | (23.0) | 524 | (22.0) | 242 | (22.5) |
| Depression only | 7201 | (17.8) | 805 | (13.2) | 322 | (12.3) | 288 | (12.1) | 105 | (9.7) |
| Stress-related only | 11059 | (27.3) | 1417 | (23.3) | 645 | (24.6) | 537 | (22.6) | 232 | (21.5) |
| Anxiety and depression | 3204 | (7.9) | 560 | (9.2) | 247 | (9.4) | 240 | (10.1) | 117 | (10.9) |
| Anxiety and stress | 3464 | (8.5) | 762 | (12.5) | 359 | (13.7) | 365 | (15.3) | 159 | (14.8) |
| Depression and stress-related | 3013 | (7.4) | 610 | (10.0) | 255 | (9.7) | 257 | (10.8) | 127 | (11.8) |
| Anxiety, depression, and stress-related | 1734 | (4.3) | 416 | (6.8) | 188 | (7.2) | 170 | (7.1) | 95 | (8.8) |
| **Number of CMD primary care visits, mean (SD) ^c^** | 4.32 | (5.67) | 5.94 | (6.50) | 5.76 | (6.54) | 6.30 | (7.40) | 7.51 | (9.74) |
| **Months since first diagnosis, mean (SD)** | 16.5 | (15.9) | 13.2 | (14.3) | 13.4 | (15.3) | 14.4 | (15.7) | 15.4 | (16.2) |
| **Prior mental disorder diagnoses (non-CMD) - primary care ^d^** |  |  |  |  |  |  |  |  |  |  |
| None | 38563 | (95.2) | 5814 | (95.5) | 2494 | (95.2) | 2280 | (95.8) | 1031 | (95.7) |
| Any | 1954 | (4.8) | 276 | (4.5) | 125 | (4.8) | 101 | (4.2) | 46 | (4.3) |
| **Prior somatic conditions - primary care ^d^** |  |  |  |  |  |  |  |  |  |  |
| None | 7705 | (19.0) | 1125 | (18.5) | 425 | (16.2) | 420 | (17.6) | 158 | (14.7) |
| Any | 32812 | (81.0) | 4965 | (81.5) | 2194 | (83.8) | 1961 | (82.4) | 919 | (85.3) |
| **Prior suicide attempts - primary care ^d^** |  |  |  |  |  |  |  |  |  |  |
| None | 40195 | (99.2) | 6050 | (99.3) | 2601 | (99.3) | 2361 | (99.2) | 1066 | (99.0) |
| Any | 322 | (0.8) | 40 | (0.7) | 18 | (0.7) | 20 | (0.8) | 11 | (1.0) |
| **Prior receipt of non-systematic therapy – primary care ^d^** |  |  |  |  |  |  |  |  |  |  |
| None | 25854 | (63.8) | 1181 | (19.4) | 1070 | (40.9) | 952 | (40.0) | 398 | (37.0) |
| Any | 14663 | (36.2) | 4909 | (80.6) | 1549 | (59.1) | 1429 | (60.0) | 679 | (63.0) |
| **Prior mental disorders diagnoses (any) - secondary care ^d^** |  |  |  |  |  |  |  |  |  |  |
| None | 31560 | (77.9) | 5004 | (82.2) | 2175 | (83.0) | 1929 | (81.0) | 862 | (80.0) |
| Any | 8957 | (22.1) | 1086 | (17.8) | 444 | (17.0) | 452 | (19.0) | 215 | (20.0) |
| **Prior suicide attempts - secondary care ^d^** |  |  |  |  |  |  |  |  |  |  |
| None | 40285 | (99.4) | 6059 | (99.5) | 2609 | (99.6) | 2373 | (99.7) | 1072 | (99.5) |
| Any | 232 | (0.6) | 31 | (0.5) | 10 | (0.4) | 8 | (0.3) | 5 | (0.5) |
| **Prior CMD-relevant psychotropic medication ^d^** |  |  |  |  |  |  |  |  |  |  |
| None | 11896 | (29.4) | 2131 | (35.0) | 1014 | (38.7) | 840 | (35.3) | 391 | (36.3) |
| Any | 28621 | (70.6) | 3959 | (65.0) | 1605 | (61.3) | 1541 | (64.7) | 686 | (63.7) |
| **Prior other psychotropic medication ^d^** |  |  |  |  |  |  |  |  |  |  |
| None | 38300 | (94.5) | 5897 | (96.8) | 2554 | (97.5) | 2295 | (96.4) | 1052 | (97.7) |
| Any | 2217 | (5.5) | 193 | (3.2) | 65 | (2.5) | 86 | (3.6) | 25 | (2.3) |

CMD: common mental disorder.

^a^ Measured during year of cohort entry.

^b^ Measured on 31^st^ December in the calendar year prior to cohort entry

^c^ Measured on cohort entry date and during two relative years (730 days) prior to cohort entry.

^d^ Measured in the two relative years (730 days) prior to cohort entry.

**Table S4. Sociodemographic and clinical characteristics of individuals diagnosed with common mental disorders in primary care who received 1-2, 3-5, 6-12, and >12 sessions of systematic psychological therapies during the two-year follow-up period (treated) and any sessions (control) during the study period for the inverse probability weighted samples**

|  | **Inverse probability weighted sample** | | | | | | | | | | **Maximum standardised difference in weighted means / proportions** |
| --- | --- | --- | --- | --- | --- | --- | --- | --- | --- | --- | --- |
|  | **Control group**  **(N=40,517)** | | **1-2 SPT sessions**  **(N=6090)** | | **3-5 SPT session**  **(N=2619)** | | **6-12 SPT sessions**  **(N=2381)** | | **>12 SPT sessions**  **(N=1077)** | |  |
|  | **N** | **(%)** | **N** | **(%)** | **N** | **(%)** | **N** | **(%)** | **N** | **(%)** |  |
| **Age (years), mean (SD) ^a^** | 41.84 | (11.3) | 41.84 | (11.4) | 41.84 | (11.2) | 41.84 | (11.2) | 41.84 | (11.1) | <0.001 |
| **Sex** |  |  |  |  |  |  |  |  |  |  |  |
| Female | 36881 | (69.4) | 36881 | (69.4) | 36881 | (69.4) | 36881 | (69.4) | 36881 | (69.4) | <0.001 |
| Male | 16228 | (30.6) | 16228 | (30.6) | 16228 | (30.6) | 16228 | (30.6) | 16228 | (30.6) | <0.001 |
| **Family situation ^b^** |  |  |  |  |  |  |  |  |  |  |  |
| Married/cohabitant without children | 6679 | (12.6) | 6679 | (12.6) | 6679 | (12.6) | 6679 | (12.6) | 6679 | (12.6) | <0.001 |
| Married/cohabitant with children | 17572 | (33.1) | 17572 | (33.1) | 17572 | (33.1) | 17572 | (33.1) | 17572 | (33.1) | <0.001 |
| Single without children | 23844 | (44.9) | 23844 | (44.9) | 23844 | (44.9) | 23844 | (44.9) | 23844 | (44.9) | <0.001 |
| Single with children | 5015 | (9.4) | 5015 | (9.4) | 5015 | (9.4) | 5015 | (9.4) | 5015 | (9.4) | <0.001 |
| **Type of residence area ^b^** |  |  |  |  |  |  |  |  |  |  |  |
| Cities | 36973 | (69.6) | 36973 | (69.6) | 36973 | (69.6) | 36973 | (69.6) | 36973 | (69.6) | <0.001 |
| Towns and suburbs | 13638 | (25.7) | 13638 | (25.7) | 13638 | (25.7) | 13638 | (25.7) | 13638 | (25.7) | <0.001 |
| Rural areas | 2498 | (4.7) | 2498 | (4.7) | 2498 | (4.7) | 2498 | (4.7) | 2498 | (4.7) | <0.001 |
| **Country of birth ^b^** |  |  |  |  |  |  |  |  |  |  |  |
| Sweden | 41467 | (78.1) | 41467 | (78.1) | 41467 | (78.1) | 41467 | (78.1) | 41467 | (78.1) | <0.001 |
| Rest of the world | 11642 | (21.9) | 11642 | (21.9) | 11642 | (21.9) | 11642 | (21.9) | 11642 | (21.9) | <0.001 |
| **Level of education ^b^** |  |  |  |  |  |  |  |  |  |  |  |
| ≤9 years | 4628 | (8.7) | 4628 | (8.7) | 4628 | (8.7) | 4628 | (8.7) | 4628 | (8.7) | <0.001 |
| 10-12 years | 21594 | (40.7) | 21594 | (40.7) | 21594 | (40.7) | 21594 | (40.7) | 21594 | (40.7) | <0.001 |
| >12 years | 26887 | (50.6) | 26887 | (50.6) | 26887 | (50.6) | 26887 | (50.6) | 26887 | (50.6) | <0.001 |
| **Days of unemployment ^b^** |  |  |  |  |  |  |  |  |  |  |  |
| None | 49510 | (93.2) | 49510 | (93.2) | 49510 | (93.2) | 49510 | (93.22) | 49510 | (93.2) | <0.001 |
| Any | 3599 | (6.8) | 3599 | (6.8) | 3599 | (6.8) | 3599 | (6.78) | 3599 | (6.8) | <0.001 |
| **CMD diagnostic profile ^c^** |  |  |  |  |  |  |  |  |  |  |  |
| Anxiety only | 13511 | (25.4) | 13511 | (25.4) | 13511 | (25.4) | 13511 | (25.4) | 13511 | (25.4) | <0.001 |
| Depression only | 8541 | (16.1) | 8541 | (16.1) | 8541 | (16.1) | 8541 | (16.1) | 8541 | (16.1) | <0.001 |
| Stress-related only | 14039 | (26.4) | 14039 | (26.4) | 14039 | (26.4) | 14039 | (26.4) | 14039 | (26.4) | <0.001 |
| Anxiety and depression | 4425 | (8.3) | 4425 | (8.3) | 4425 | (8.3) | 4425 | (8.3) | 4425 | (8.3) | <0.001 |
| Anxiety and stress | 5381 | (10.1) | 5381 | (10.1) | 5381 | (10.1) | 5381 | (10.1) | 5381 | (10.1) | <0.001 |
| Depression and stress-related | 4421 | (8.3) | 4421 | (8.3) | 4421 | (8.3) | 4421 | (8.3) | 4421 | (8.3) | <0.001 |
| Anxiety, depression, and stress-related | 2792 | (5.3) | 2792 | (5.3) | 2792 | (5.3) | 2792 | (5.3) | 2792 | (5.3) | <0.001 |
| **Number of CMD primary care visits, mean (SD) ^c^** | 5.21 | (9.54) | 5.21 | (5.92) | 5.21 | (5.51) | 5.21 | (5.36) | 5.21 | (5.41) | <0.001 |
| **Months since first diagnosis, mean (SD)** | 15.99 | (15.50) | 15.99 | (16.88) | 15.99 | (16.94) | 15.99 | (16.86) | 15.99 | (17.49) | <0.001 |
| **Prior mental disorder diagnoses (non-CMD) - primary care ^d^** |  |  |  |  |  |  |  |  |  |  |  |
| None | 50618 | (95.3) | 50618 | (95.3) | 50618 | (95.3) | 50618 | (95.3) | 50618 | (95.3) | <0.001 |
| Any | 2491 | (4.7) | 2491 | (4.7) | 2491 | (4.7) | 2491 | (4.7) | 2491 | (4.7) | <0.001 |
| **Prior somatic conditions - primary care ^d^** |  |  |  |  |  |  |  |  |  |  |  |
| None | 9850 | (18.5) | 9850 | (18.5) | 9850 | (18.5) | 9850 | (18.5) | 9850 | (18.5) | <0.001 |
| Any | 43259 | (81.5) | 43259 | (81.5) | 43259 | (81.5) | 43259 | (81.5) | 43259 | (81.5) | <0.001 |
| **Prior suicide attempts - primary care ^d^** |  |  |  |  |  |  |  |  |  |  |  |
| None | 52681 | (99.2) | 52681 | (99.2) | 52681 | (99.2) | 52681 | (99.2) | 52681 | (99.2) | <0.001 |
| Any | 429 | (0.8) | 429 | (0.8) | 429 | (0.8) | 429 | (0.8) | 429 | (0.8) | <0.001 |
| **Prior receipt of non-systematic therapy – primary care ^d^** |  |  |  |  |  |  |  |  |  |  |  |
| None | 29167 | (54.9) | 29167 | (54.9) | 29167 | (54.9) | 29167 | (54.9) | 29167 | (54.9) | <0.001 |
| Any | 23943 | (45.1) | 23943 | (45.1) | 23943 | (45.1) | 23943 | (45.1) | 23943 | (45.1) | <0.001 |
| **Prior mental disorders diagnoses (any) - secondary care ^d^** |  |  |  |  |  |  |  |  |  |  |  |
| None | 41702 | (78.5) | 41702 | (78.5) | 41702 | (78.5) | 41702 | (78.5) | 41702 | (78.5) | <0.001 |
| Any | 11408 | (21.5) | 11408 | (21.5) | 11408 | (21.5) | 11408 | (21.5) | 11408 | (21.5) | <0.001 |
| **Prior suicide attempts - secondary care ^d^** |  |  |  |  |  |  |  |  |  |  |  |
| None | 52830 | (99.5) | 52830 | (99.5) | 52830 | (99.5) | 52830 | (99.5) | 52830 | (99.5) | <0.001 |
| Any | 280 | (0.5) | 280 | (0.5) | 280 | (0.5) | 280 | (0.5) | 280 | (0.5) | <0.001 |
| **Prior CMD-relevant psychotropic medication ^d^** |  |  |  |  |  |  |  |  |  |  |  |
| None | 16712 | (31.5) | 16712 | (31.5) | 16712 | (31.5) | 16712 | (31.5) | 16712 | (31.5) | <0.001 |
| Any | 36397 | (68.5) | 36397 | (68.5) | 36397 | (68.5) | 36397 | (68.5) | 36397 | (68.5) | <0.001 |
| **Prior other psychotropic medication ^d^** |  |  |  |  |  |  |  |  |  |  |  |
| None | 50500 | (95.1) | 50500 | (95.1) | 50500 | (95.1) | 50500 | (95.1) | 50500 | (95.1) | <0.001 |
| Any | 2609 | (4.9) | 2609 | (4.9) | 2609 | (4.9) | 2609 | (4.9) | 2609 | (4.9) | <0.001 |

CMD: common mental disorder, ESS: effective sample size.

^a^ Measured during year of cohort entry.

^b^ Measured on 31^st^ December in the calendar year prior to cohort entry

^c^ Measured on cohort entry date and during two relative years (730 days) prior to cohort entry.

^d^ Measured in the two relative years (730 days) prior to cohort entry.

**Table** **S5. Number (%) of individuals having >14, >30, and > 90 net sickness absence days at each six-month time interval relative to the six months prior to t0 among individuals with common mental disorders who received 1-2, 3-5, 6-12, and >12 sessions of systematic psychological therapy (treated group) and those who did not (control group)**

|  | **Crude sample** | | | | | **Inverse probability weighted sample** | | | | |
| --- | --- | --- | --- | --- | --- | --- | --- | --- | --- | --- |
| Time interval ^a^ | **Control** | **1-2 Sessions** | **3-5 Sessions** | **6-12 Sessions** | **>12 Sessions** | **Control** | **1-2 Sessions** | **3-5 Sessions** | **6-12 Sessions** | **>12 Sessions** |
| **SA > 14 days** |  |  |  |  |  |  |  |  |  |  |
| t-18 | 5791 (14.3%) | 687 (11.3%) | 317 (12.1%) | 284 (11.9%) | 153 (14.2%) | 7879 (14.8%) | 6426 (12.1%) | 6469 (12.2%) | 5633 (10.6%) | 5912 (11.1%) |
| t-12 | 6741 (16.6%) | 803 (13.2%) | 343 (13.1%) | 365 (15.3%) | 192 (17.8%) | 9300 (17.5%) | 7283 (13.7%) | 6681 (12.6%) | 7187 (13.5%) | 7344 (13.8%) |
| t-6 | 8367 (20.7%) | 1224 (20.1%) | 490 (18.7%) | 479 (20.1%) | 217 (20.1%) | 11647 (21.9%) | 10157 (19.1%) | 9543 (18.0%) | 9400 (17.7%) | 9333 (17.6%) |
| t-0 | 11342 (28.0%) | 2263 (37.2%) | 952 (36.3%) | 828 (34.8%) | 381 (35.4%) | 15854 (29.9%) | 18389 (34.6%) | 18327 (34.5%) | 17170 (32.3%) | 17509 (33.0%) |
| t6 | 8907 (22.0%) | 1538 (25.3%) | 889 (33.9%) | 832 (34.9%) | 421 (39.1%) | 12139 (22.9%) | 15592 (29.4%) | 17878 (33.7%) | 18185 (34.2%) | 19932 (37.5%) |
| t12 | 6436 (15.9%) | 909 (14.9%) | 480 (18.3%) | 532 (22.3%) | 333 (30.9%) | 8688 (16.4%) | 8682 (16.4%) | 9958 (18.7%) | 11743 (22.1%) | 17343 (32.7%) |
| t18 | 5429 (13.4%) | 713 (11.7%) | 422 (16.1%) | 453 (19.0%) | 276 (25.6%) | 7284 (13.7%) | 7027 (13.2%) | 8829 (16.6%) | 9905 (18.7%) | 13126 (24.7%) |
| t24 | 5215 (12.9%) | 735 (12.1%) | 407 (15.5%) | 402 (16.9%) | 245 (22.7%) | 6983 (13.2%) | 7070 (13.3%) | 8496 (16.0%) | 8644 (16.3%) | 11240 (21.2%) |
| **SA > 30 days** |  |  |  |  |  |  |  |  |  |  |
| t-18 | 4457 (11.0%) | 522 (8.6%) | 215 (8.2%) | 197 (8.3%) | 114 (10.6%) | 6142 (11.6%) | 5037 (9.5%) | 4388 (8.3%) | 3734 (7.0%) | 4202 (7.9%) |
| t-12 | 5220 (12.9%) | 590 (9.7%) | 262 (10.0%) | 263 (11.0%) | 145 (13.5%) | 7317 (13.8%) | 5362 (10.1%) | 4954 (9.3%) | 4864 (9.2%) | 5635 (10.6%) |
| t-6 | 6409 (15.8%) | 921 (15.1%) | 367 (14.0%) | 377 (15.8%) | 178 (16.5%) | 9043 (17.0%) | 7631 (14.4%) | 7088 (13.3%) | 7168 (13.5%) | 7657 (14.4%) |
| t-0 | 8510 (21.0%) | 1818 (29.9%) | 703 (26.8%) | 621 (26.1%) | 300 (27.9%) | 12127 (22.8%) | 13671 (25.7%) | 13128 (24.7%) | 12335 (23.2%) | 13836 (26.1%) |
| t6 | 6979 (17.2%) | 1188 (19.5%) | 734 (28.0%) | 713 (29.9%) | 358 (33.2%) | 9564 (18.0%) | 11865 (22.3%) | 14922 (28.1%) | 15823 (29.8%) | 17362 (32.7%) |
| t12 | 4995 (12.3%) | 720 (11.8%) | 390 (14.9%) | 431 (18.1%) | 287 (26.6%) | 6801 (12.8%) | 6845 (12.9%) | 8028 (15.1%) | 9571 (18.0%) | 15308 (28.8%) |
| t18 | 4137 (10.2%) | 540 (8.9%) | 328 (12.5%) | 355 (14.9%) | 243 (22.6%) | 5583 (10.5%) | 5318 (10.0%) | 7019 (13.2%) | 7786 (14.7%) | 11689 (22.0%) |
| t24 | 3889 (9.6%) | 527 (8.7%) | 317 (12.1%) | 316 (13.3%) | 202 (18.8%) | 5263 (9.9%) | 5014 (9.5%) | 6740 (12.7%) | 6660 (12.6%) | 9645 (18.2%) |
| **SA > 90 days** |  |  |  |  |  |  |  |  |  |  |
| t-18 | 2119 (5.2%) | 251 (4.1%) | 95 (3.6%) | 103 (4.3%) | 57 (5.3%) | 3044 (5.7%) | 2698 (5.1%) | 1965 (3.7%) | 2038 (3.8%) | 2134 (4.0%) |
| t-12 | 2399 (5.9%) | 249 (4.1%) | 116 (4.4%) | 125 (5.2%) | 74 (6.9%) | 3473 (6.5%) | 2397 (4.5%) | 1976 (3.7%) | 2232 (4.2%) | 2971 (5.6%) |
| t-6 | 2843 (7.0%) | 396 (6.5%) | 167 (6.4%) | 190 (8.0%) | 90 (8.4%) | 4145 (7.8%) | 3066 (5.8%) | 3087 (5.8%) | 3451 (6.5%) | 3648 (6.9%) |
| t-0 | 3544 (8.7%) | 778 (12.8%) | 289 (11.0%) | 265 (11.1%) | 125 (11.6%) | 5111 (9.6%) | 5241 (9.9%) | 5120 (9.6%) | 4843 (9.1%) | 5055 (9.5%) |
| t6 | 3422 (8.4%) | 592 (9.7%) | 365 (13.9%) | 374 (15.7%) | 227 (21.1%) | 4757 (9.0%) | 5911 (11.1%) | 7296 (13.7%) | 8299 (15.6%) | 11292 (21.3%) |
| t12 | 2472 (6.1%) | 362 (5.9%) | 208 (7.9%) | 211 (8.9%) | 156 (14.5%) | 3391 (6.4%) | 3617 (6.8%) | 4116 (7.7%) | 4688 (8.8%) | 7978 (15.0%) |
| t18 | 2094 (5.2%) | 263 (4.3%) | 160 (6.1%) | 182 (7.6%) | 136 (12.6%) | 2827 (5.3%) | 2712 (5.1%) | 3479 (6.6%) | 3969 (7.5%) | 7179 (13.5%) |
| t24 | 1863 (4.6%) | 243 (4.0%) | 145 (5.5%) | 153 (6.4%) | 125 (11.6%) | 2512 (4.7%) | 2404 (4.5%) | 3001 (5.7%) | 3178 (6.0%) | 5959 (11.2%) |

SA, sickness absence. ^a^ Time interval corresponds to 6-month intervals (182 days) measured relative to the date of cohort entry (treated group: cohort entry date corresponds to date of first observed systematic psychological therapy; control group: cohort entry date corresponds to randomly selected date from grid of possible start dates based on year and month of first observed CMD visit). ^b^ Inverse probability weights derived using covariate balancing propensity score weighting methods, incorporating all sociodemographic and clinical covariates.
